# Supplementary material for: Combinatorial entropy behaviour leads to range selective binding in ligand-receptor interactions
Source: Nat Commun. 2020 Sep 24;11:4836. doi: 10.1038/s41467-020-18603-5 (PMC7515919; doi:10.1038/s41467-020-18603-5)
Supplement: Supplementary file 1 — Supplementary Information [file 41467_2020_18603_MOESM1_ESM.pdf]

Supplementary Information for "Combinatorial entropy behaviour leads to range selective binding in ligand-receptor interactions", Meng et al

SUPPLEMENTARY METHODS I

Homogeneity of ligands surface distribution in our polymersomes. In fitting the experimental data, we assume that the grafting density of ligands on the polymersomes is homogeneous. This is only correct if the copolymers used for self-assembling the polymersomes are well-mixed. As reported in Refs.[1] and [2], the thermodynamic tendency of block copolymers to phase separate and generate a non-homogeneous distribution of domains within the vesicles surface is evident when different polymers are used to form polymersomes. This phenomenon requires a certain period of time to have complete segregation of the two phases and it is affected from the storage conditions that clearly impact the kinetic of the process, as reported in Ref.[1]. The result can be then easily detected through electron microscopy techniques that reveal discontinuities in the topology of these polymeric nanovesicles. In this work, the polymers (identical in terms of chemical nature: Angiopep-2-PEG-PDPA, Cy5-PEG-PDPA and PEG-PDPA) and the protocol we have used to prepare them indeed allow to obtain well-dispersed ligands within the polymersomes without creating dissimilarities in the vesicle surface as confirmed by Transmisison Electron Microscopy (TEM) analysis (see Supplementary Figure 3 and Supplementary Figure 4 here). The samples were stored at 4°C and were used immediately after their preparation, a temperature at which phase separation, even if thermodynamically possible, would be kinetically blocked.

SUPPLEMENTARY METHODS II

Stability of the polymersomes over time and reproducibility across different batches. We report here a study using DLS on different samples to prove both the stability of our particles over time as well as their reproducibility across different batches, see Supplementary Figure 1 and 2 for reference. More specifically, in Supplementary Figure 1, we report the raw correlograms taken via DLS on our samples.

In Supplementary Figure 2, we summarise the results of a study comparing different batches of particles or the same batch at different time-points. Specifically, in panel A “Time stability” we report the DLS number distribution of the samples used for this study at the time when the experiments have been performed (AP-PEGPDPA psomes t=0, blue solid

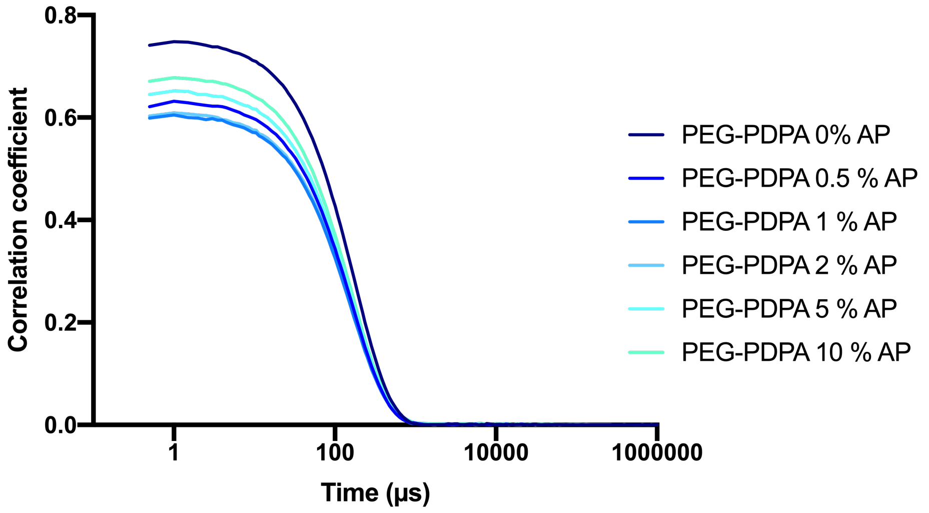

a)

Supplementary Figure 1. *Correlograms from our Dynamic Light Scattering experiments, from which stability of the colloidal suspensions used in our experiments can be easily observed (all raw data from the DLS are available online in raw form in the DataFile file).*

lines) and 1 year later (AP-PEGPDPA psomes 1 year, blue dashed lines). In panel B "Batch reproducibility", we report the DLS characterisation of the samples used for the experiments in this work (AP-PEGPDPA psomes t=0, blue solid lines) and a second independent batch of ligand-and fluorophore-conjugated polymersomes immediately after the sample preparation (AP-PEGPDPA independent batch, green solid lines). In order to prove that the presence of the ligands or the fluorophore in our polymersome formulations does not affect their size, in panel C "Batch reproducibility among pristine and functionalized psomes" we report a comparison between the number distribution of the ligand- and fluorophore-conjugated polymersomes used for the experiments in this study (AP-PEGPDPA psomes t=0, blue solid lines) and pristine PEG-PDPA polymersomes immediately after the preparation (pristine psomes t=0, black solid line) and 1 year after their preparation (pristine psomes 1 year, red solid line). As the reader can appreciate, no significant differences can be observed among different batches and over long periods of time.

SUPPLEMENTARY METHODS III

Polydispersity and its effect on range-selectivity .

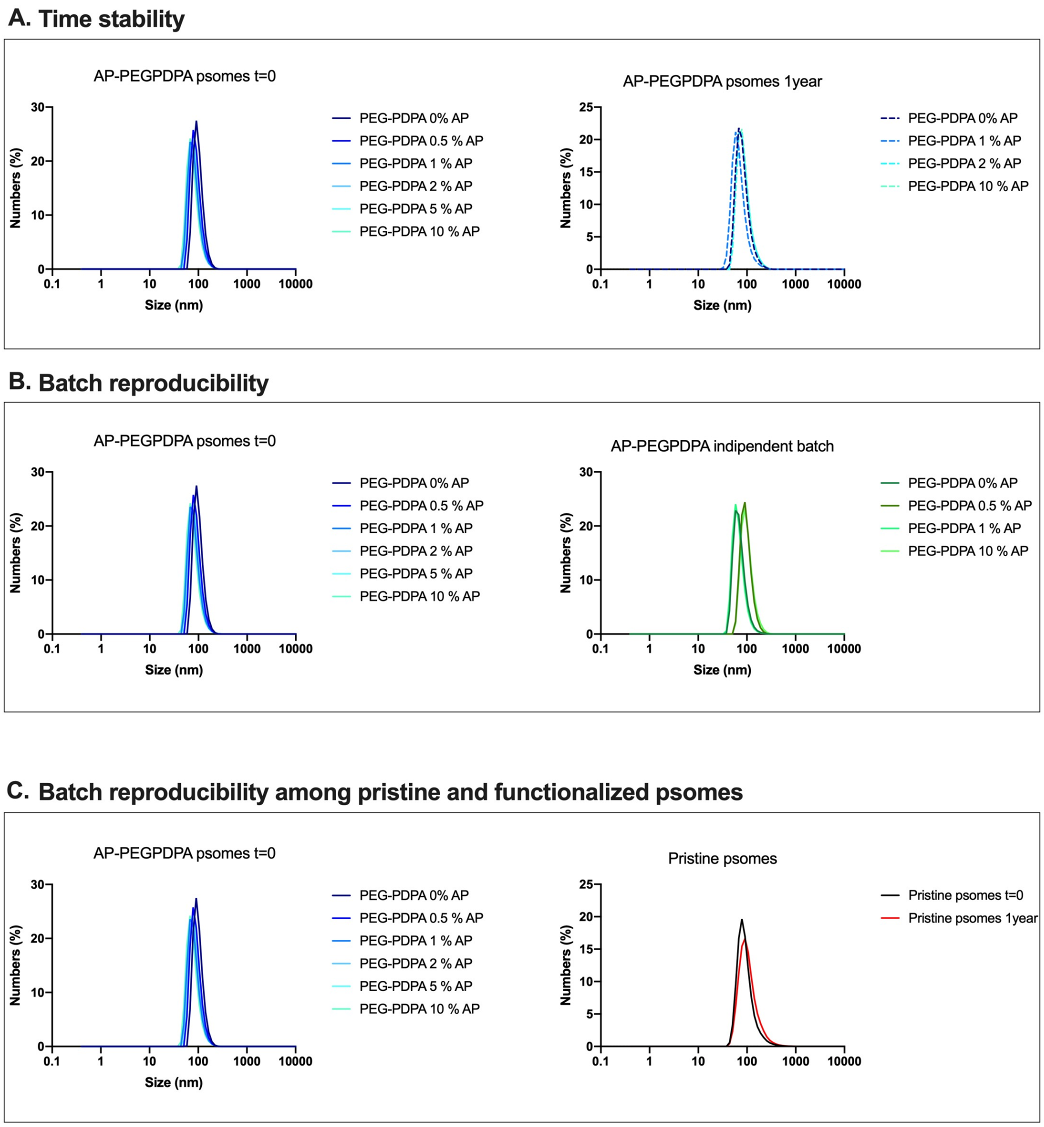

Supplementary Figure 2. *Time-stability of the functionalised polymersomes (abbreviated as psomes in the graphs). (Details in the main text of Supplementary Note II).*

In order to gauge polydispersity in our sample, Transmission Electron Microscopy was used together with a Matlab algorithm for image analysis (a link to the GitHub repository with the Matlab script used to compute the PDI is available at the following link: https://github.com/GabrieleMarchello/PDI). Briefly, the algorithm adopted was comprised of various steps, as we present here. Firstly, the image was scanned in a raster way and divided into small patches (i.e. areas of pixels) partially overlapping. Then, the mean intensity value of these patches was subtracted from the intensity of the patches, in order to compensate the uneven illumination of the image. The current implementation of the algorithm extracted 128x128 square patches, extracted every 2 pixels in both directions [3].

Furthermore, the image was filtered by using a Gaussian filter 2 pixels wide. This filter smoothed the details of the image down, reducing significantly the amount of misleading information on the image, in such a way to make the particle identification more robust [4]. In order to simplify the particle identification step, the edges (i.e. the sharp variations of brightness) in the image were computed, creating a mask with the profiles of the imaged elements. The edges were computed by applying the Canny method [5]. At this point, the circular elements in the image were identified, saving only the elements with the two main dimensions differing at most by the 20% of their value.

Once all the particles were identified in the image, their diameter is computed as the mean value of their two main dimensions. Finally, the mean values of all the diameters $\bar{\mu}$ and their standard deviation $\bar{\sigma}$ are computed in order to calculate the PDI as shown in the following equation.

$$PDI = (\mu/\sigma)^2 \tag{1}$$

Representative TEM images and the size distribution of the polymersomes obtained from all the aggregate TEM data are reported in Supplementary Figure 3 here.

A question that might arise is what is the effect of polydispersity on range selectivity. Whereas the polydispersity of the system affects the exact quantitative details of the binding curve, the qualitative non-monotonic behaviour that we dubbed range selectivity is not affected. That this is the case can be deduced by calculating the average binding probability given a certain number distribution for our sample, i.e.

$$\langle\theta\rangle = \int_0^\infty \theta(R)P(R). \tag{2}$$

In order to show the effect of including polydispersity, we plot in Supplementary Figure 8 the value of $\langle\theta\rangle$ calculated for a mono-disperse dispersion as well as a log-normal distribution for the size in our samples (consistent with the fact that $R > 0$):

$$P(R) = \frac{1}{\sqrt{2\pi}\sigma R}\exp(\frac{(\ln R - \mu)^2}{2\sigma^2}). \tag{3}$$

Note that the mean $\bar{R} = \langle R\rangle$ and variance $\bar{R^2} = \langle R^2\rangle$ - $\langle R\rangle^2$ in our samples as measured by Transmission electron microscopy can be used to derive μ and σ for the lognormal distribution as:

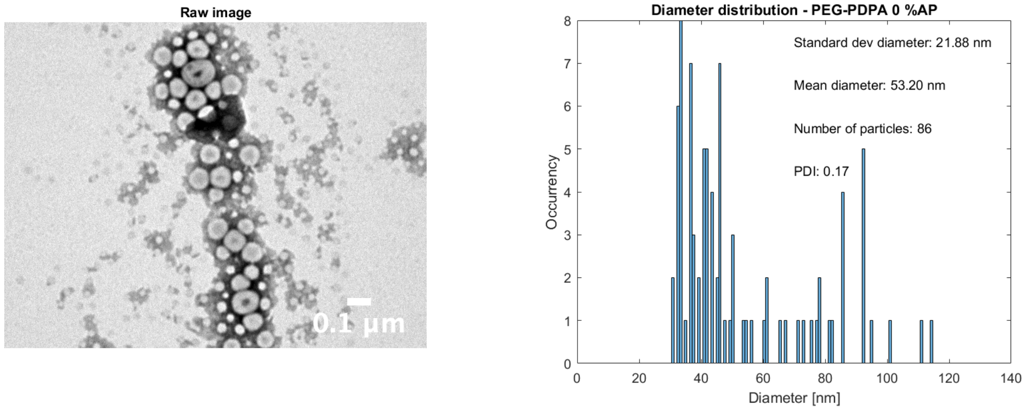

a)

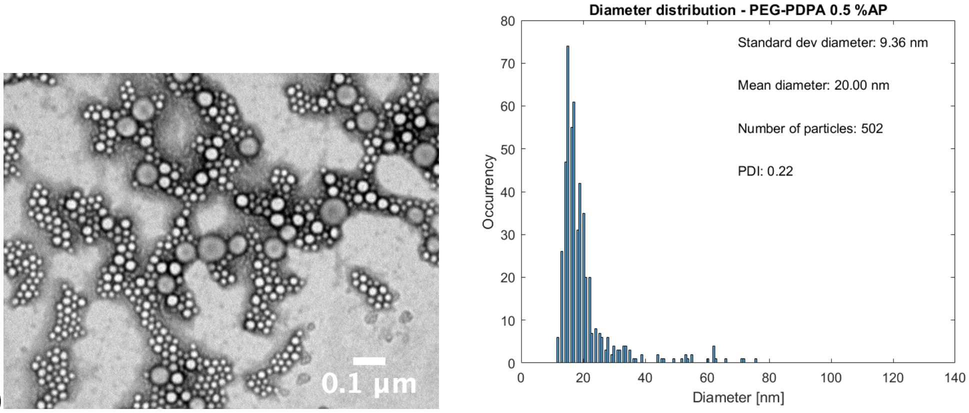

b)

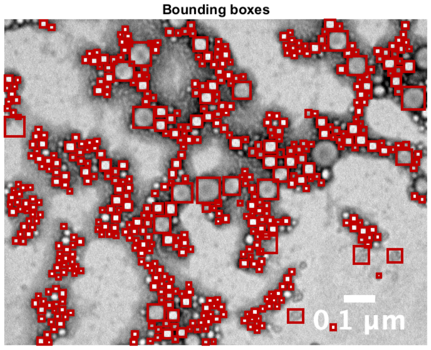

c)

Supplementary Figure 3. *a),b) Representative TEM images (left image) and the corresponding size-distribution of the particle (right image) at different ligand loadings of* 0% *and* 0.5%*, respectively. c) A representative image with the result of applying the algorithm used to analyse the size distribution on the TEM images on a sample with grafting density* 0.5%*. All raw data are available online in raw form from inside the DataFile archive*

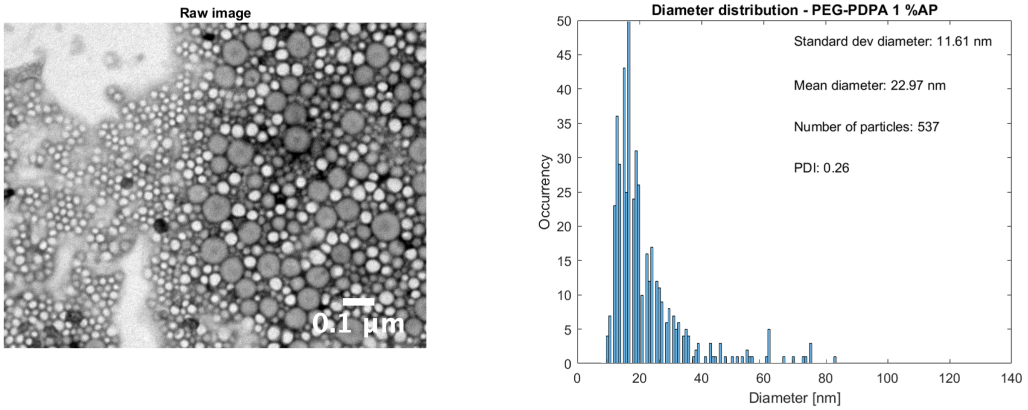

c)

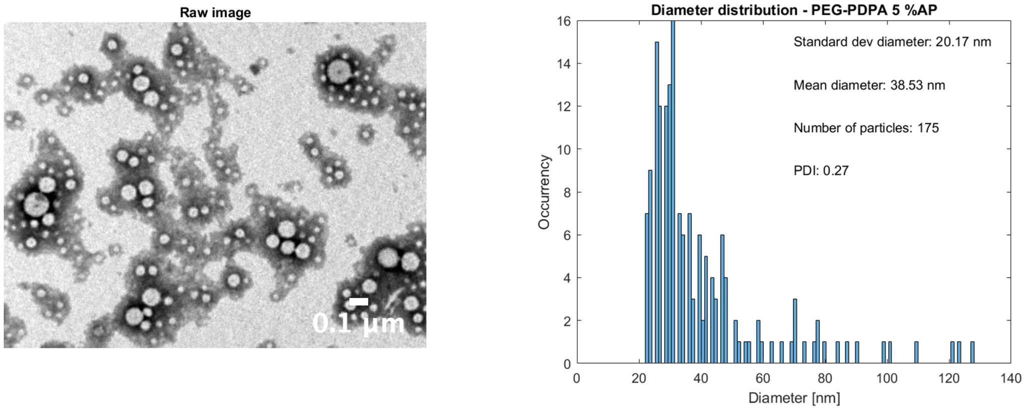

d)

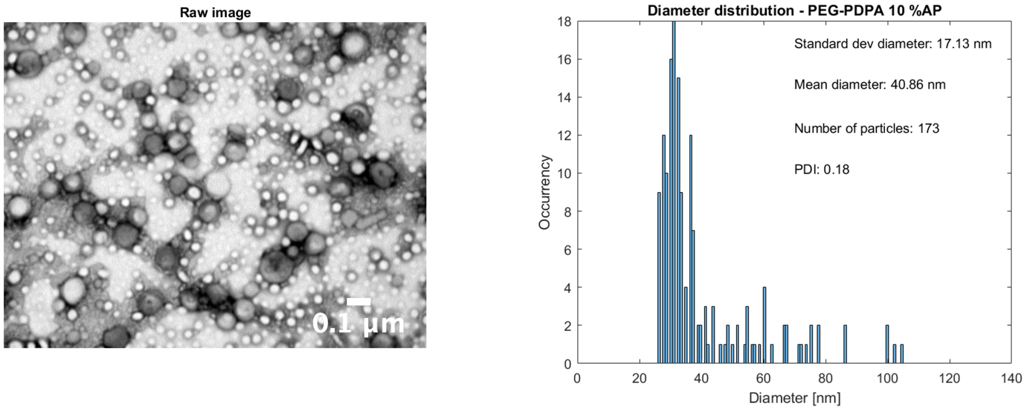

e)

Supplementary Figure 4. *c)-e) as in Supplementary Figure 3 but for ligands loading equal to* $1, 5 and 10\%$*, respectively. All raw data are available online in raw form from inside the DataFile.zip file*

$$\mu = \log\left(\frac{\bar{R}}{\sqrt{\frac{\bar{R^2}}{\bar{R}^2}+1}}\right) \tag{4}$$

and

$$\sigma^2 = \log\left(\frac{\bar{R^2}}{\bar{R}^2} + 1\right) \tag{5}$$

For the sake of providing a relevant example, we chose here to use a mean radius of 23 nm and a mean-square root deviation of 12 nm, as for the case of 1% functionalisation in our polymersomes. All other values characterising the polymersome and the ligand-receptor pair are taken to be exactly those in our experimental system. As it can be seen, the effect of polydispersity is minimal. Moreover, it should be appreciated how the occurrence of range selectivity, even for this relatively elevated values of polydispersity, is again not much affected and its non-monotonic behaviour is robust with respect to it (see Supplementary Figure 5), as we argued above.

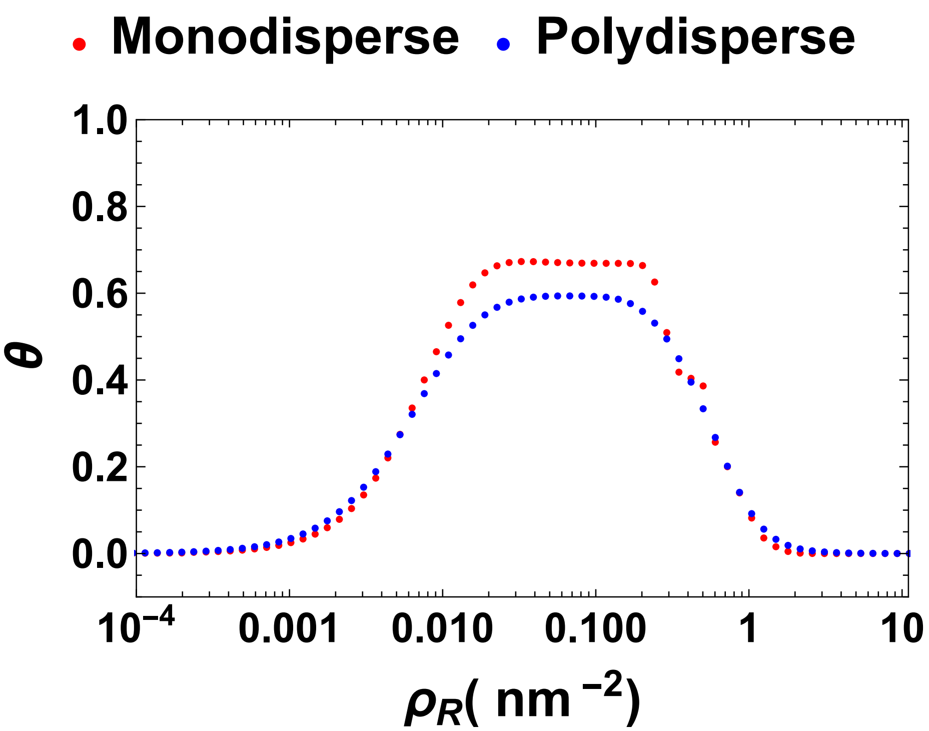

Supplementary Figure 5. *Comparison of the binding probability of a mono vs polydisperse system. In the polydisperse system, we use a mean and variance equal to that of our sample at 1% ligand loading, which gives a value of the polydispersity index of approximately 0.25, representative to that of all our samples. As it can be seen, range selectivity is preserved and the effect of polydispersity is simply to slightly smooth the adsorption curve.*

SUPPLEMENTARY METHODS III

Polymersomes adsorption on the membrane. We show here in Supplementary Figure 6 a representative set of images, whose analysis was used to establish the adsorption data reported in Fig.4a) in the main text. As it is also qualitatively clear by a naked-eye analysis, due to the non-monotonic nature of the adsorption probability in range-selective systems the red-fluorescent signal increases going from polymersomes with 0% to 1% ligands loading, and then decreases again at 10% loading. Note that all confocal microscopy files from which experimental data have been calculated are available online in raw form from the DataFile.zip file.

SUPPLEMENTARY NOTES I

Assumed Binding Geometry for fitting. In our calculation for fitting the experimental data, we need to calculate the number of ligands that can interact with the receptors on the surface, i.e. all those residing within a region A_{int}, see Supplementary Figure 7. We do this by assuming that every ligand whose grafting point is at a maximum distance $d = 2R_g$ from the surface, R_g being the ligand gyration radius, can bind receptors (note that this value is the most probable value of the end-to-end distance in a Gaussian chain). In practice, ligands that are much farther than that will have to stretch too much, and their effective bond energy will become very large (see e.g. [6] for a full treatment of the distance-dependent single-bond energy), or in other words the bond very weak, providing a negligible contribution to binding. Similarly, ligands and receptors away from this region (or its projection on the binding surface $A_{\text{int}}^{\text{surf}}$ for the case of receptors) are barely confined between the nanoparticle and the cell surface, thus also providing a negligible contribution to the repulsive energy. Overall, this provides the following formulas for the interactive area on the nanoparticle and on the surface, $A_{\text{int}}^{\text{np}}$ and $A_{\text{int}}^{\text{surf}}$, respectively:

$$A_{\text{int}}^{\text{np}} = 2\pi R R_g \tag{6}$$

$$A_{\text{int}}^{\text{surf}} = \pi\left[R^2 - (R - R_g)^2\right], \tag{7}$$

where R and R_g are the nanoparticle radius and the gyration radius of the ligand, respectively, please see Supplementary Figure 7 for reference,

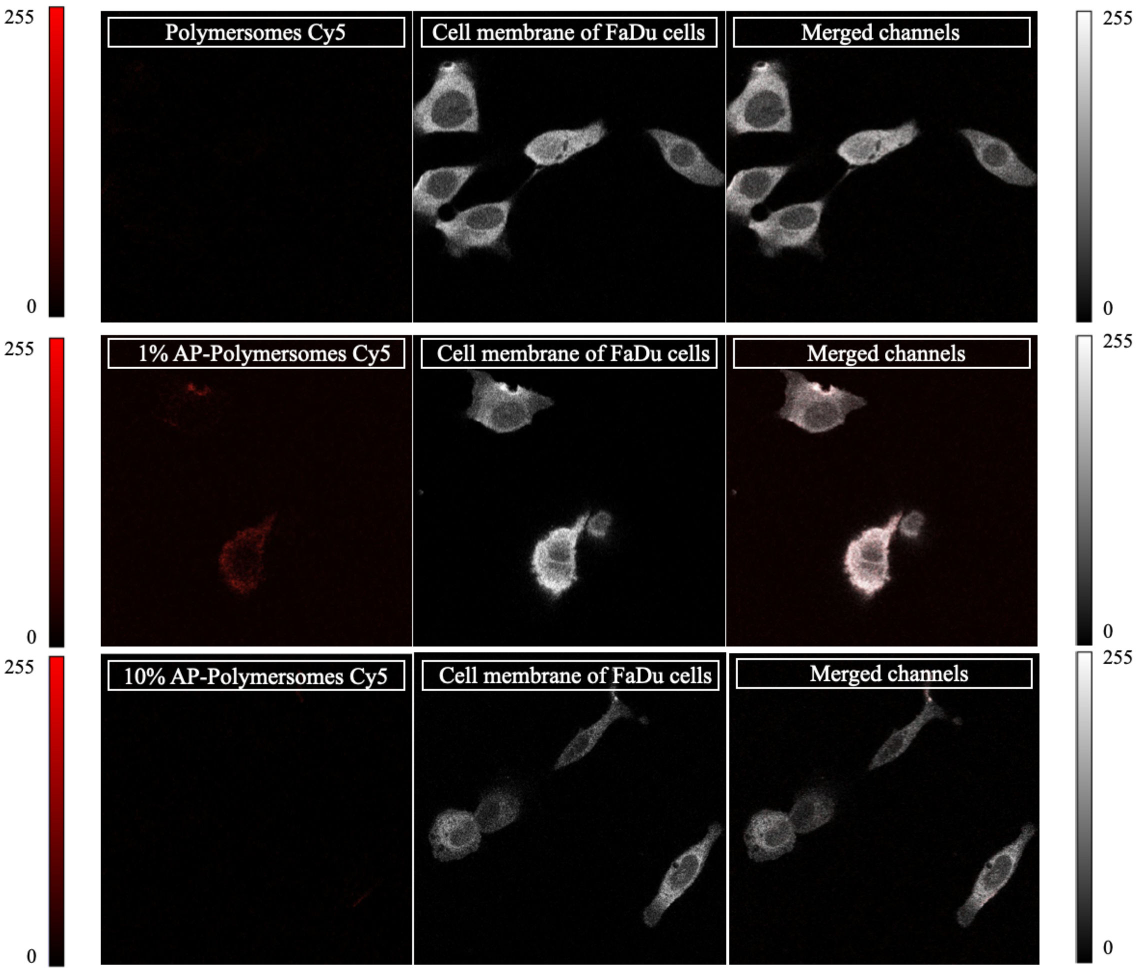

Supplementary Figure 6. *Confocal microscopy micrographs of polymersome binding to the cellular membrane after 1 h of incubation at 37°C. From left to right, Cy5-labelled polymersomes, CellMask Green-labelled cellular membrane of FaDu and colocalization of polymersomes and cellular membrane. From top to bottom, the series of images correspond to pristine (i.e., 0%, non-functionalised polymersomes), 1% and 10% Angiopep-2-functionalised polymersomes, respectively. A clear variation in the fluorescent signal can be seen among the samples from the Cy5-labelled polymersomes channel (in red). This variation is associated to the different amount of polymersomes adsorbed on the cellular membrane as a function of the Angiopep-2 functionalisation level. In particular, there is initially a clear increase in the fluorescent signal between 0% and 1%, which then drops again at 10% loading due to the non-monotonic adsorption behaviour, see also Fig.4a) in the main text.*

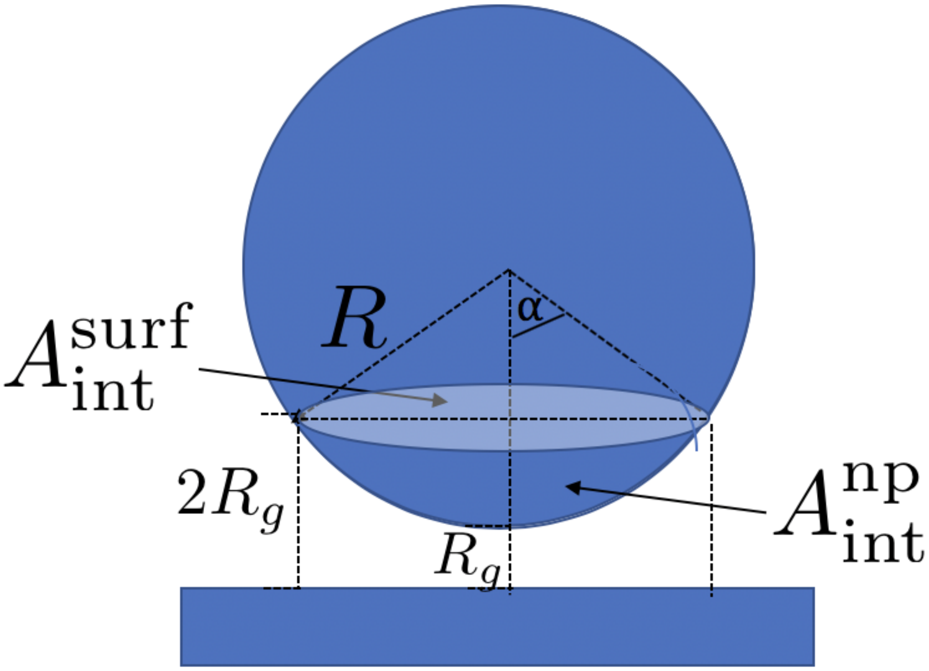

Supplementary Figure 7. *Binding geometry for the nanoparticle. Nanoparticles are assumed to bind at a distance* $d = R_g$ *from the surface. As an approximation, all ligands whose grafting point is at a maximum distance of* $d_{\mathrm{max}} = 2R_g$ *from the surface are able to bind receptors. This approximation leads to the formulas in Supplementary Equations 6 and* (7) *for the interaction area and is only used to estimate the number of interacting ligands from the experimentally available grafting density for the plot in Fig.4 of the main text. Note, however, that the observation of range selectivity does not depend on this approximations.*

SUPPLEMENTARY NOTES II

Calculation of the bound partition function q. The calculation of the bound partition function depends somehow on the definition of the bound state. Similarly to what has been done in [7], we can start by assuming that any particle whose distance to the surface is below a certain value z_0, defined as the value at which bonds can form, can be considered as bound. As we shall see, however, the exact choice of this distance is not important. Let us call $\Delta F(z)$ the total free-energy as a function of distance z between the nanoparticle and the surface, measured with respect to a reference state where no bonds are possible (i.e., the particle is in the bulk far away from the binding surface). The bound partition function (q in Eq.(**??**) in the main text) can be defined as [7]:

$$q = A_{\text{site}} \int_0^{z_0} \exp(-\beta \Delta F(z)) dz, \quad (8)$$

where $A_{\text{site}} = \pi R^2$ is the area occupied by a single particle and excluded to others, R being the nanoparticle radius. Now if we look at the form of $\Delta F(z)$, this will have a minimum in a region of width $\approx R_g$ around $z \approx R_g$, thus allowing us to use a saddle-point approximation for calculating the integral in Eq.(8). The minimum is expected to be at around Rg for the following reason: for $z \ll R_g$, both the ligands and the receptors are highly compressed (against the binding surface or the nanoparticle's brush, respectively), quickly increasing the repulsive contribution F_{rep} and thus F_{tot}. For $z \gg R_g$, whereas the repulsive contribution quickly drops to zero, ligands must instead stretch a lot to bind the receptors, leading to very weak bonds and in turns to a small value (in magnitude), of the binding free-energy. For this reason, the minimum will be around $z = R_g$, where $F_{\text{rep}} \approx 0$ but F_{att} gives a sizeable (negative) contribution. We note here that we are not the first to use this approximation, see e.g. [7], which gives good agreement with detailed molecular simulations.

SUPPLEMENTARY NOTES III

A mean-field approximation to the radial binding scenario. Eq.7 in the main text provides a mean-field approximation to the radial binding scenario. This approximation can be arrived at in the following way. Consider N_L ligands, each of which can bind N_R receptors with a single-bond energy ΔG. In a mean-field approximation, ligands are considered independent from each other and thus the total partition function Q can be just written as the product of the single-ligand partition function q, i.e:

$$Q = q^{N_L} \quad (9)$$

$$q = \left[1 + N_R \exp(-\beta \Delta G)\right], \quad (10)$$

where the term 1 in q corresponds to the ligand in the unbound state and the second term corresponds to each of the N_R states where the ligand is bound to one of the N_R different receptors available. One thus arrives at the following approximation for the attractive part of the free energy as:

$$\beta F_{\mathrm{att}} = -\log Q^{N_L} = -N_L \log\left[1 + N_R \exp(-\beta \Delta G)\right]. \tag{11}$$

Note that some authors define Q as the sum over all states where at least a single bond is present, and thus add a term of -1 from Q to remove the contribution from the state where no bonds at all a present [8]. Here instead we use a definition for the partition function so that it is the attractive free-energy F_{att} rather than Q that goes to zero when no bonds can be made (formally, when $\Delta G \to +\infty$) [9].
By assuming a mean-field approximation for the receptors, i.e. assuming they are independent from each other, the symmetric formula where N_L is substituted with N_R, and vice-versa, is obtained. The reason the mean-field approximation works well in the regime $N_L \ll N_R$ (or vice-versa, when receptors are considered) is because in this regime the ligands are almost independent. Whereas for a more in-depth and general discussion we refer the reader to Refs.[6, 9], we consider here for simplicity the case of two ligands, each of which can bind the same N_R receptors. Let us thus consider the conditional probabilities $p_{1,\bar{2}}$ and $p_{1,2}$ of ligand 1 being bound, given that ligand 2 is not, and that ligand 1 is bound given that ligand 2 also is. Using Bayes theorem, we can write these as:

$$p_{1,\bar{2}} = \frac{Q_{1\&\bar{2}}}{Q_{\bar{2}}} = \frac{N_R \chi}{1 + N_R \chi} \tag{12}$$

$$p_{1,2} = \frac{Q_{1\&2}}{Q_2} = \frac{N_R(N_R - 1)\chi}{1 + N_R\chi + N_R(N_R-1)\chi}, \tag{13}$$

where we defined $\chi = \exp(-\beta\Delta G)$ and $Q_{1\&\bar{2}}$ and $Q_{1\&2}$ are the partial partition functions summing over all contributions where ligand 1 is bound, and 2 is not, or where both are bound, respectively. For independent ligands, one would have that the two conditional probabilities must be the same, or in other words what happens to ligand 2 is irrelevant for the state of ligand 1. If we take the limit for a large number of receptors and use to make an approximation via a Taylor expansion in the variable $\alpha = N_R^{-1}$ we have both probabilities are the same to first order in α, $p_{1,\bar{2}} \approx p_{1,\bar{2}} \approx 1 - \chi\alpha$, hence ligands behave independently as suggested, and a mean-field approximation becomes a good approximation.

SUPPLEMENTARY NOTES IV

Super-selectivity parameter in our system In order to evaluate how sharp is the response in binding to a variation in the number of receptors, we report in Supplementary Figure 8 the equivalent of the curves in Fig.2 of the main text as a log-log plot. Note that the derivative of this curve is the so-called super-selectivity parameter α [8]. However, because of the non-monotonic behaviour of the bound partition function and the corresponding binding probability, the typical interpretation of α as in standard (i.e., monotonic) multivalent adsorbing systems is not valid. More precisely, $\alpha > 1$ does not necessarily mean a super-linear response, nor can α be interpreted as the Hill-exponent to be used to fit multivalent binding adsorption curves.

SUPPLEMENTARY NOTES V

Estimating the kinetics for binding from the brush. In order to give a rough estimate for the binding kinetics and how this is influenced from the brush, we use a simple Debye-Smoluchowsky description to determine the average time at which a nanoparticle reaches the surface of a cell, taken to be the average distance at which a ligand-receptor bond is made [10]. As we are only interested in an order of magnitude estimate, we treat both the cell and the nanoparticles as floating spheres in solution with radii $R = R_{\rm np} + h$ ($R_{\rm np}$ being the bare nanoparticle size and h the average height of its protective polymer brush) and $R_{\rm cell}$, respectively. The interaction (free-)energy is constant (and set to zero) as long as the surface-to-surface distance is larger than $R + R_{\rm cell}$ and equal to $F_{\rm rep}$, the total repulsive force due receptors and ligands (see Eq.4–6 in the main text) for a distance $R_{\rm np} + R_g < d << R$, at which the contact is considered to occur. Note that in this way we are over-estimating repulsion, since we are assuming that its value is actually constant in this region whereas in reality it will increase continuously from 0 to this value. As a result, we expect to obtain a timescale larger than the actual one.

Under the previous assumptions, the average time it takes two particles under this interaction free-energy to meet is: [10]:

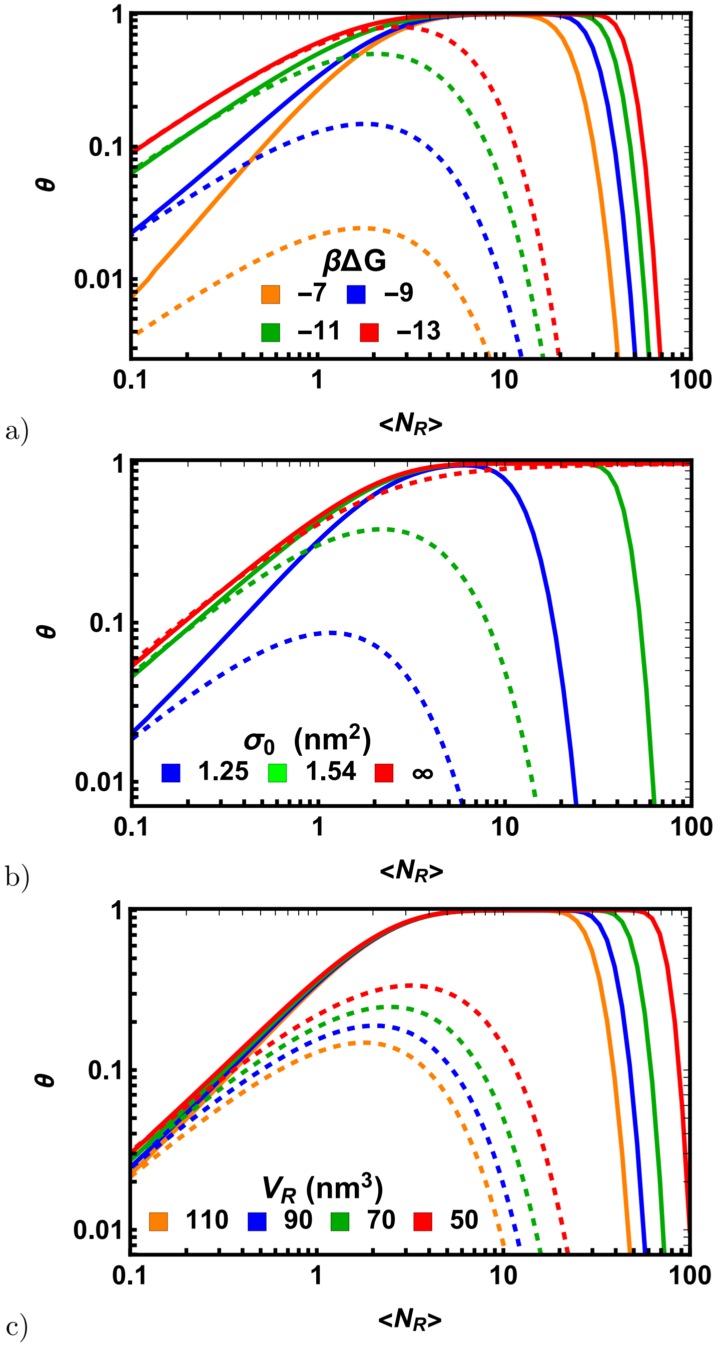

Supplementary Figure 8. *Binding probability vs number of receptors as a log-log plot for the same systems and combination of parameters as in Fig.2 in the main text.*

$$\tau = \tau_\infty + \tau_b \tag{14}$$

$$\tau_\infty = \frac{1}{z 4\pi D(R_{np} + R_{\text{cell}} + h)} \tag{15}$$

$$\tau_b = \frac{\exp(\beta F_{\text{rep}})}{z4\pi D} \frac{(h - R_g)}{(R_{np} + R_{\text{cell}} + h)(R_{np} + R_{\text{cell}} + R_g)}, \tag{16}$$

where z is the concentration of the nanoparticles in solution. Within this description, we can interpret τ_{infty} the time for the cell to get in contact with the outer part of the nanoparticle's brush and τ_b the time required to compress it by the value necessary to reach the ligand buried below it. Furthermore, the effective diffusion coefficient $D = D_{\text{cell}} + D_{\text{np}}$, where D_{np} and D_{cell} are the diffusion coefficient of a cell and nanoparticle, respectively, and we further use Einstein's relation to calculate their value in water, i.e.:

$$D_x = \frac{k_{\text{B}}T}{6\pi R_x \eta}, \tag{17}$$

$\eta \approx 8.9 \cdot 10^{-4}$ Pa s being the viscosity of water. Considering the size of a cell is about 3 orders of magnitude larger than any of the characteristics lengthscales describing the polymersomes, i.e. $R_{\text{cell}} \gg R_{\text{np}}, h, R_g$, we can further simplify $D \approx D_{\text{np}}$ and $R_{\text{cell}}+R_{\text{np}}+R_g \approx R_{\text{cell}}$, thus giving:

$$\tau_\infty = \frac{3\eta\beta R}{2zR_{\text{cell}}} \tag{18}$$

$$\tau_b = \tau_\infty \exp(\beta F_{\text{rep}}) \frac{h - R_g}{R_{\text{cell}}} \tag{19}$$

which, substituting the values for our system $h = 8\,\text{nm}$, $R_g = 3\,\text{nm}$, and $F_{\text{rep}} = 9.6$ (corresponding to the maximum value, achieved with the highest ligands loading), $R_{\text{cell}} \approx 1\mu\text{m}$ gives an impingement time of 10^{-1}s, much shorter than the timescale of our experiments (1h). It is noticed that for our system and within our assumptions τ is dominated by τ_b (compared to $\tau_\infty \approx 10^{-2}$s) and overcoming the repulsive force is the rate limiting step as long as $F_{\text{rep}} > 5k_{\text{B}}T$. Generally speaking, one should expect kinetics consideration to come into play if the adsorption measurements are taken at a time $t_{\text{exp}} < \tau$.

SUPPLEMENTARY NOTES VI

Deriving an optimal number of ligands for multivalent constructs to display

maximum binding strength. In order to derive Equation 8 in the main text, we work in the regime where $N_R \ll N_L$, where the growth of the attractive part of the free energy is logarithmic as a function of the number of ligands and we expect the total adsorption energy F_{tot} to start to increase after reaching its minimum, thus corresponding to the maximum binding strength. In this case, the total binding energy as a function of the number of ligands and receptors is, assuming for the ligands a linear repulsive contribution:

$$\beta F_{\text{tot}} = -N_R \ln\left[1 + N_L \exp(-\beta \Delta G)\right] + A N_R + B N_L \tag{20}$$

from which the minimum, corresponding to the maximum binding strength, can be easily calculated by imposing $N_L^{\text{optimal}} : \frac{\partial \beta F_{\text{tot}}}{\partial N_L}|_{N_{\text{L}}=N_{\text{L}}^{\text{optimal}}} = 0$ leading to:

$$N_L^{\text{optimal}} = \frac{N_R}{B} - \frac{1}{\chi}, \tag{21}$$

where again $\chi = \exp(-\beta \Delta G)$ can be interpreted as the bond strength, or simply a rescaled version of the ligand-receptor binding constant, since $\chi = K_{\text{bind}}/v_0$, v_0 being the ligand-receptor binding volume (more precisely, a measure of the change in the molecular partition function of the bound ligand-receptor pair [6]). A few interesting predictions can be made based on this formula. The first is that for weak enough bonds ($\chi \to 0$), the value of $N_{\text{L}}^{\text{optimal}}$ can be negative. Since for $N_L > N_L^{\text{optimal}}$ the derivative of F_{tot} with respect to N_{L} is positive, this means that in this case the total binding energy is a monotonically increasing function of the number of ligands for any physical value, hence adding ligands would only decrease the overall binding strength. The same expression also shows that even for very strong ligands ($\chi \to \infty$), although as long as at least $N_{\text{L}} > N_{\text{R}}$, the optimal number of ligands is still finite and depends on the number of receptors as well as the strength of the repulsion, i.e. $N_L^{\text{optimal}} = \frac{N_R}{B}$.

SUPPLEMENTARY REFERENCES

---

[1] Caterina LoPresti, Marzia Massignani, Christine Fernyhough, Adam Blanazs, Anthony J Ryan, Jeppe Madsen, Nicholas J Warren, Steven P Armes, Andrew L Lewis, Somyot Chi-

rasatitsin, et al. Controlling polymersome surface topology at the nanoscale by membrane confined polymer/polymer phase separation. ACS nano, 5(3):1775–1784, 2011.

[2] Lorena Ruiz-Pérez, Lea Messager, Jens Gaitzsch, Adrian Joseph, Ludovico Sutto, Francesco Luigi Gervasio, and Giuseppe Battaglia. Molecular engineering of polymersome surface topology. Science Advances, 2(4), 2016. doi:10.1126/sciadv.1500948. URL https://advances.sciencemag.org/content/2/4/e1500948.

[3] J Alex Stark. Adaptive image contrast enhancement using generalizations of histogram equalization. IEEE Transactions on image processing, 9(5):889–896, 2000.

[4] Johannes PF D'Haeyer. Gaussian filtering of images: A regularization approach. Signal Processing, 18(2):169–181, 1989.

[5] John Canny. A computational approach to edge detection. IEEE Transactions on pattern analysis and machine intelligence, (6):679–698, 1986.

[6] P. Varilly, S. Angioletti-Uberti, B. M. Mognetti, and D. Frenkel. A general theory of dna-mediated and other valence-limited colloidal interactions. The Journal of Chemical Physics, 137:094108–094122, 2012. doi:http://arxiv.org/abs/1205.6921.

[7] Tine Curk, Jure Dobnikar, and Daan Frenkel. Optimal multivalent targeting of membranes with many distinct receptors. Proceedings of the National Academy of Sciences, 114(28): 7210–7215, 2017.

[8] Francisco J Martinez-Veracoechea and Daan Frenkel. Designing super selectivity in multivalent nano-particle binding. Proceedings of the National Academy of Sciences, 108(27):10963–10968, 2011.

[9] S. Angioletti-Uberti, P. Varilly, B. M. Mognetti, A.V. Tkachenko, and D. Frenkel. Communication: A simple analytical formula for the free energy of ligand-receptor-mediated interactions. The Journal of Chemical Physics, 138:021102–021106, 2013.

[10] Stefano Angioletti-Uberti, Yan Lu, Matthias Ballauff, and Joachim Dzubiella. Theory of solvation-controlled reactions in stimuli-responsive nanoreactors. The Journal of Physical Chemistry C, 119(27):15723–15730, 2015.
