# Supplementary figures and images for: Combinatorial entropy behaviour leads to range selective binding in ligand-receptor interactions
Source: Nat Commun. 2020 Sep 24;11:4836. doi: 10.1038/s41467-020-18603-5 (PMC7515919; doi:10.1038/s41467-020-18603-5)

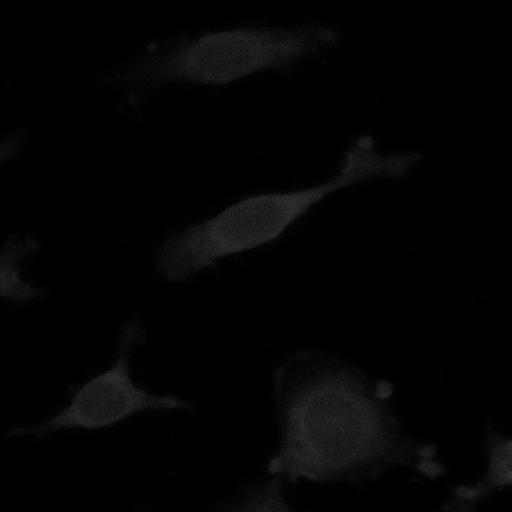

Supplement: Supplementary file 3 — Source Data [file 41467_2020_18603_MOESM3_ESM.zip › Correlograms_and_data_analysis/Avg intensity cells.tiff]

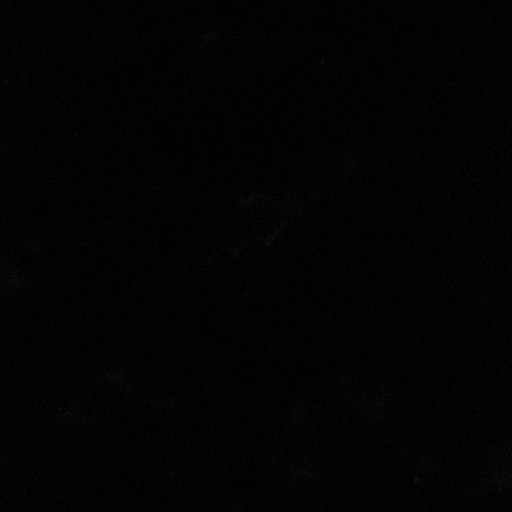

Supplement: Supplementary file 3 — Source Data [file 41467_2020_18603_MOESM3_ESM.zip › Correlograms_and_data_analysis/Avg intensity psomes.tif]

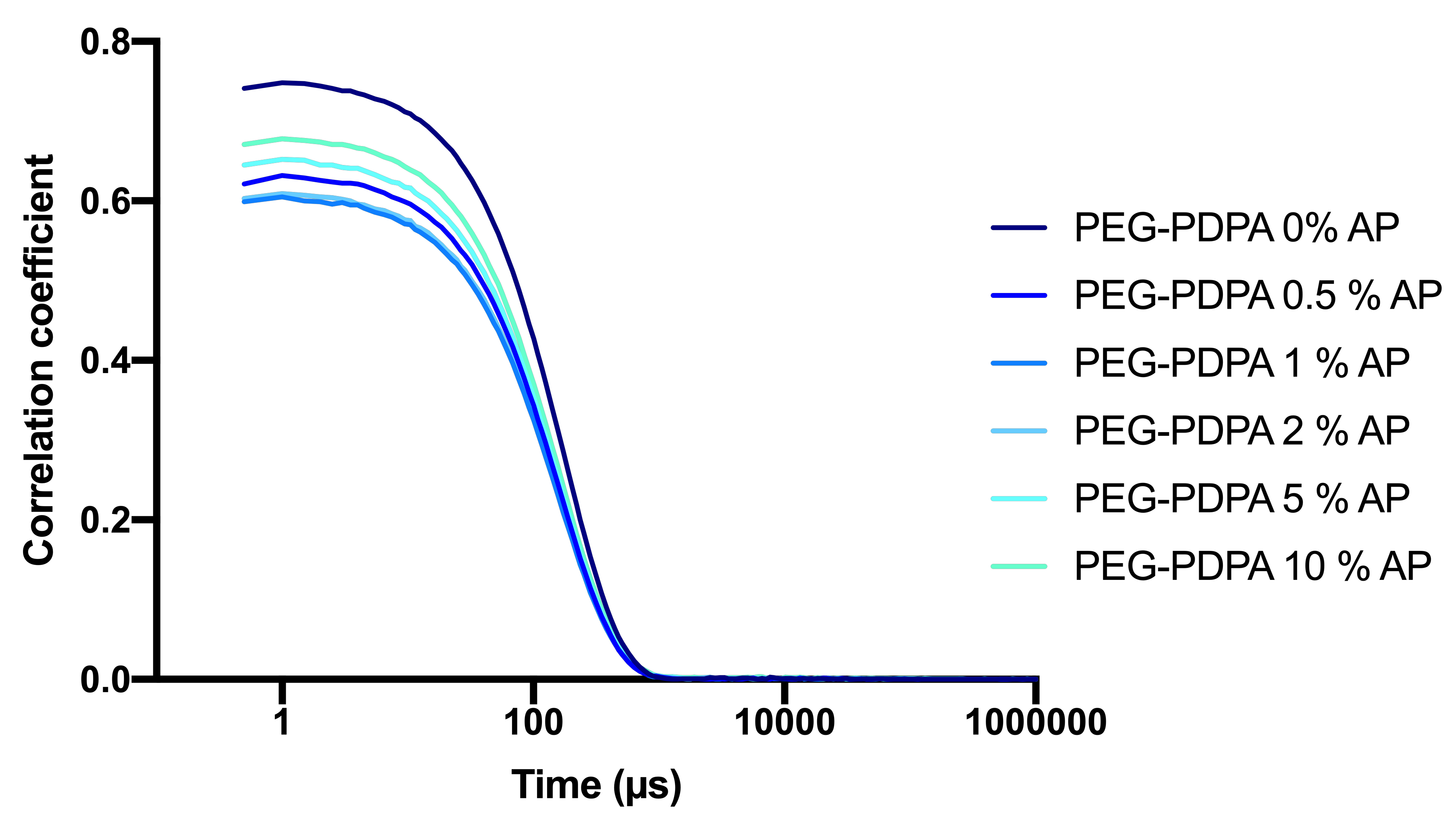

Supplement: Supplementary file 3 — Source Data [file 41467_2020_18603_MOESM3_ESM.zip › Correlograms_and_data_analysis/Correlograms.tiff]

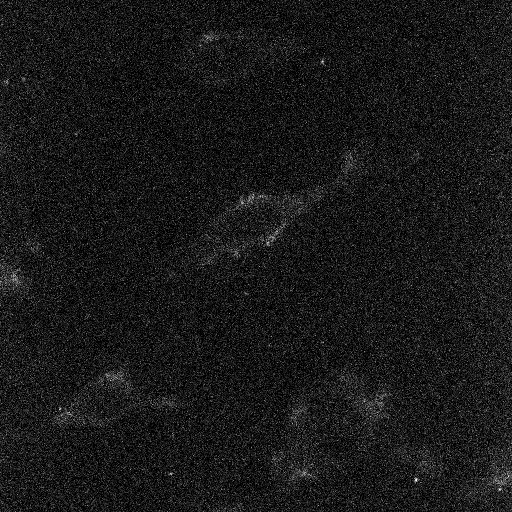

Supplement: Supplementary file 3 — Source Data [file 41467_2020_18603_MOESM3_ESM.zip › Correlograms_and_data_analysis/sum intensity psomes.jpg]

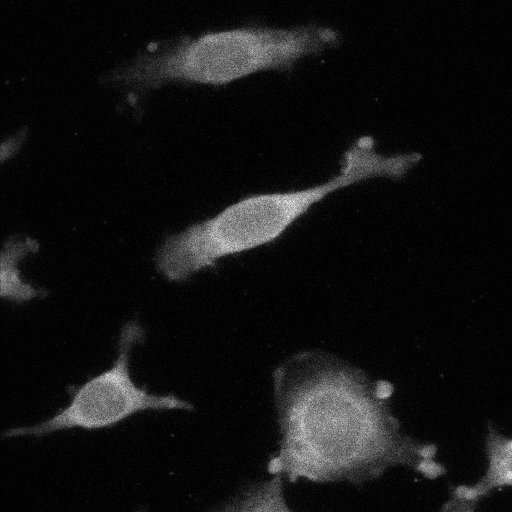

Supplement: Supplementary file 3 — Source Data [file 41467_2020_18603_MOESM3_ESM.zip › Correlograms_and_data_analysis/sum intensity.jpg]

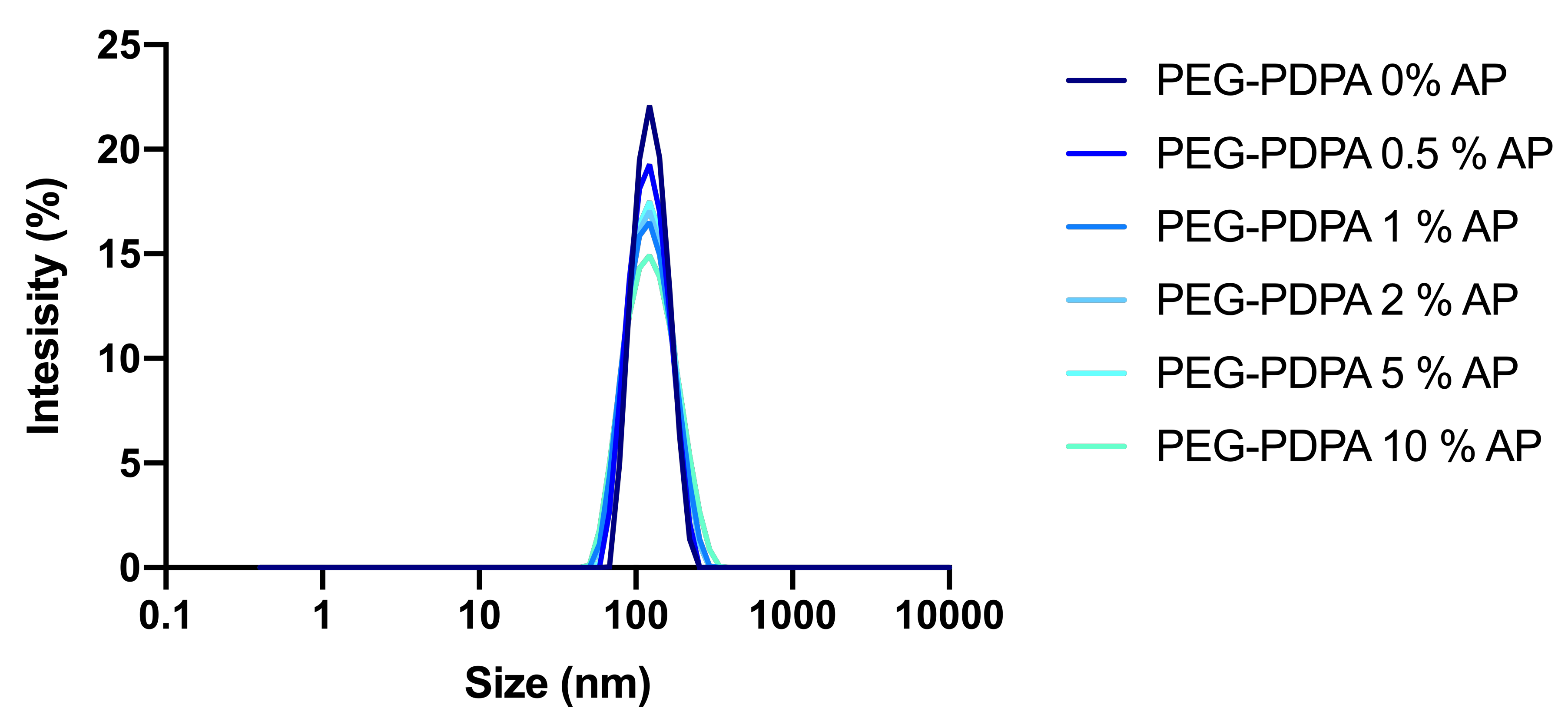

Supplement: Supplementary file 3 — Source Data [file 41467_2020_18603_MOESM3_ESM.zip › DLS_Plots_3_samples/DLS Intensity.tiff]

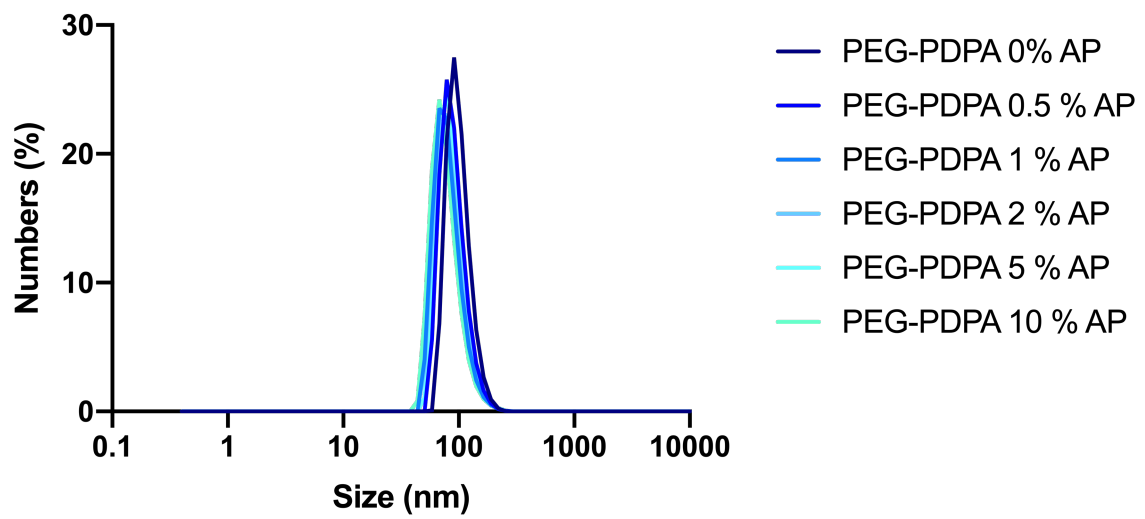

Supplement: Supplementary file 3 — Source Data [file 41467_2020_18603_MOESM3_ESM.zip › DLS_Plots_3_samples/DLS numbers.pdf]

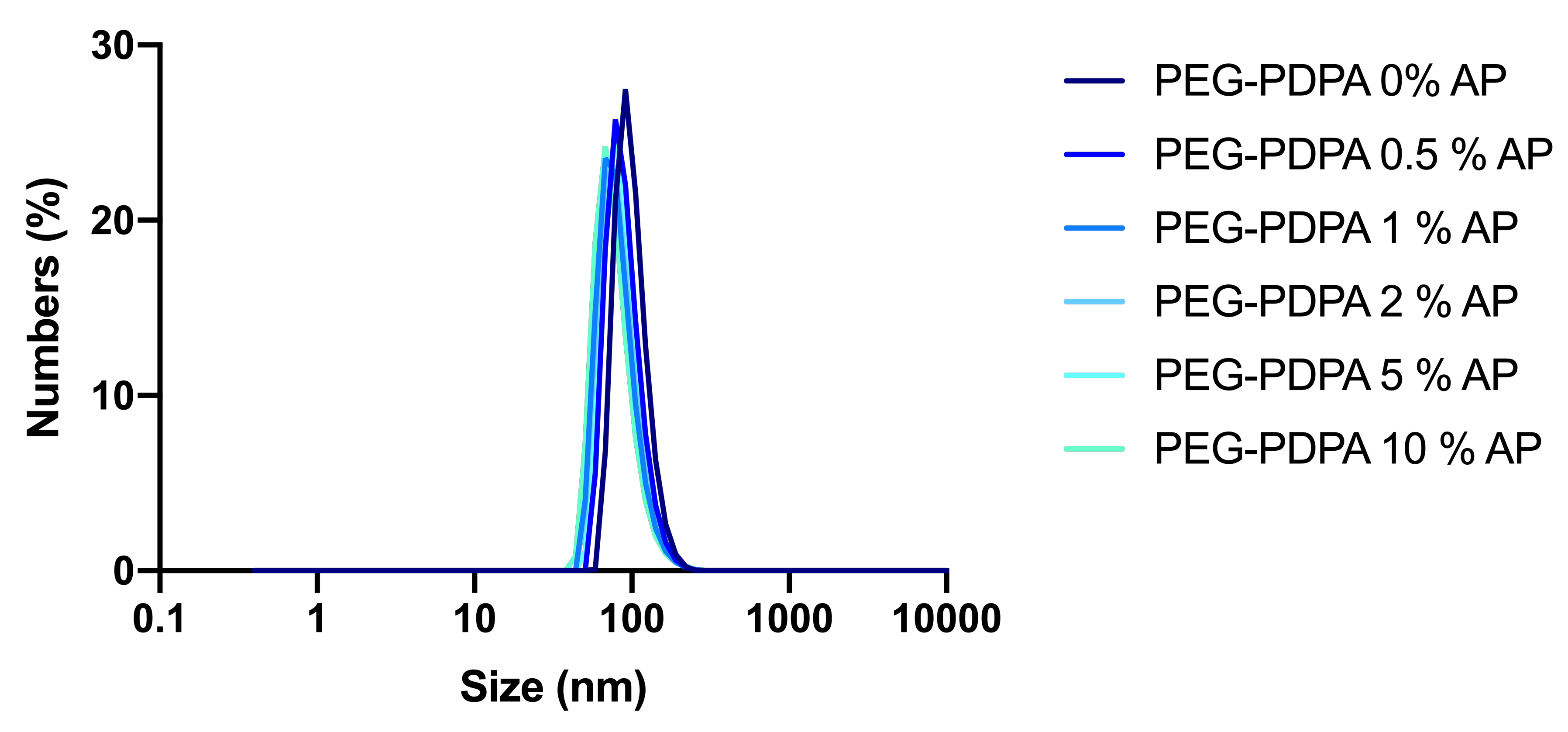

Supplement: Supplementary file 3 — Source Data [file 41467_2020_18603_MOESM3_ESM.zip › DLS_Plots_3_samples/DLS numbers.tiff]

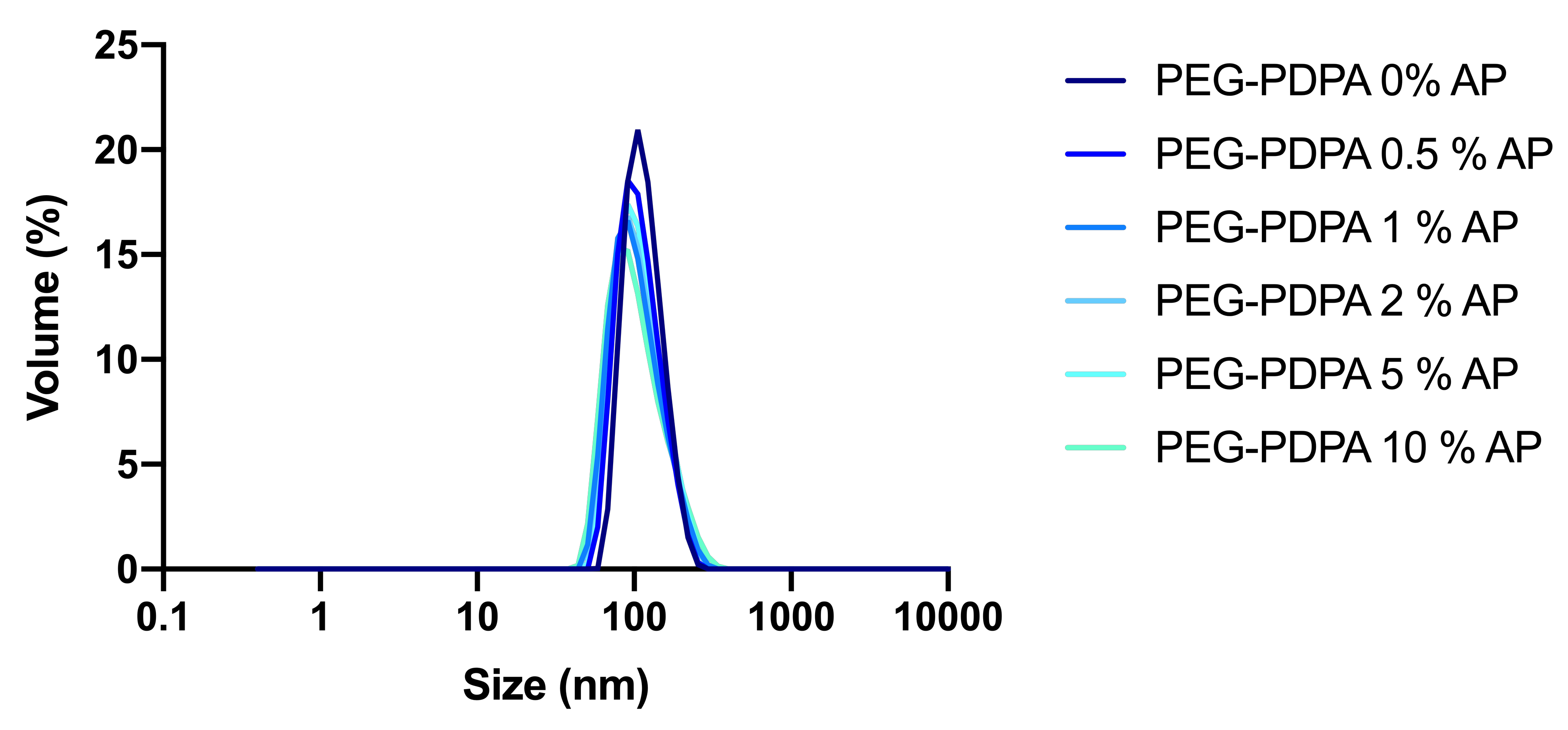

Supplement: Supplementary file 3 — Source Data [file 41467_2020_18603_MOESM3_ESM.zip › DLS_Plots_3_samples/DLS Volume.tiff]

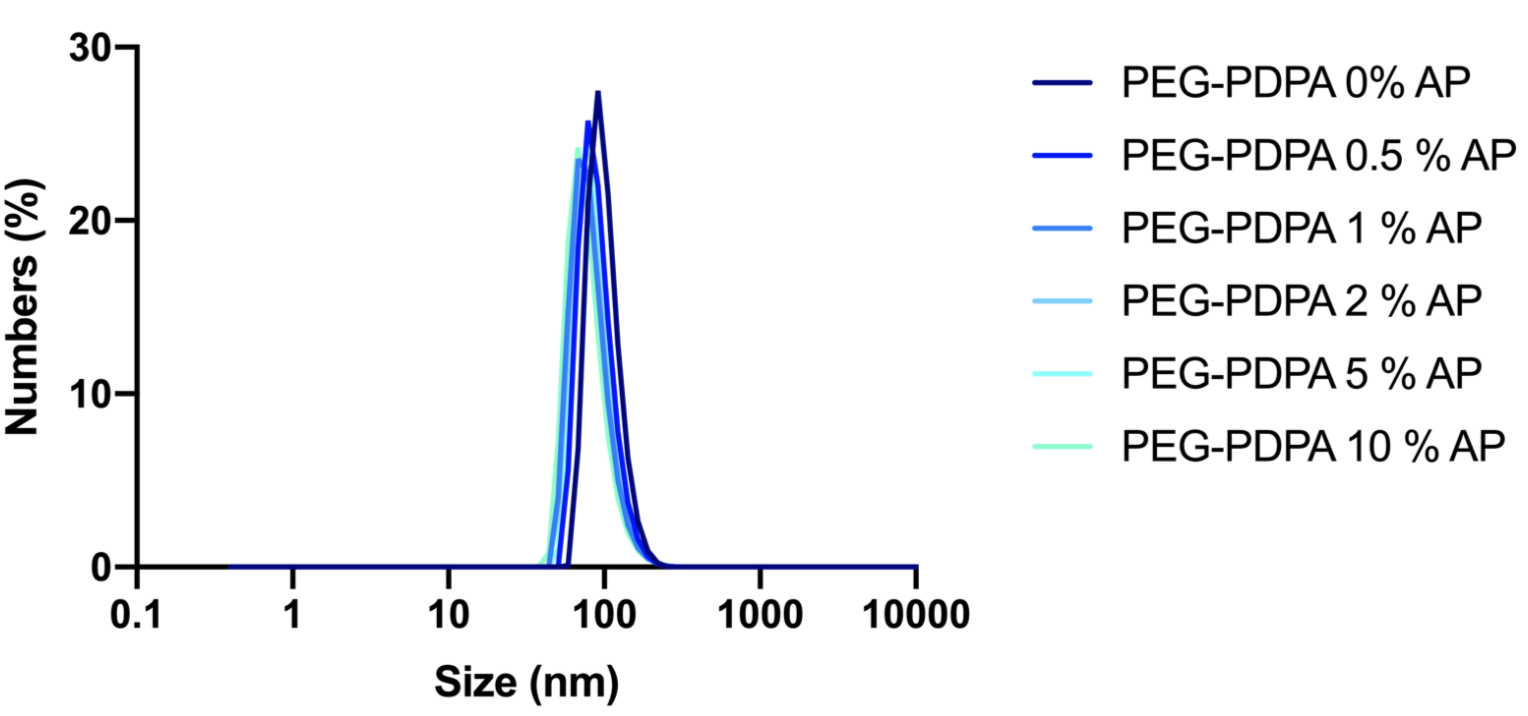

Supplement: Supplementary file 3 — Source Data [file 41467_2020_18603_MOESM3_ESM.zip › DLS_Plots_3_samples/DLS_numbers.png]

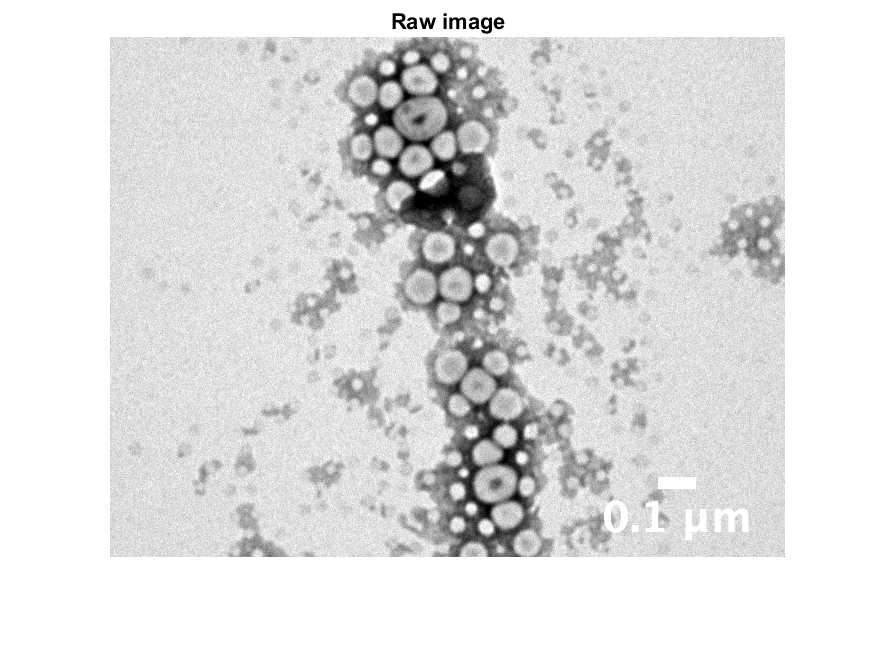

Supplement: Supplementary file 3 — Source Data [file 41467_2020_18603_MOESM3_ESM.zip › DLS_Plots_3_samples/Raw0.png]

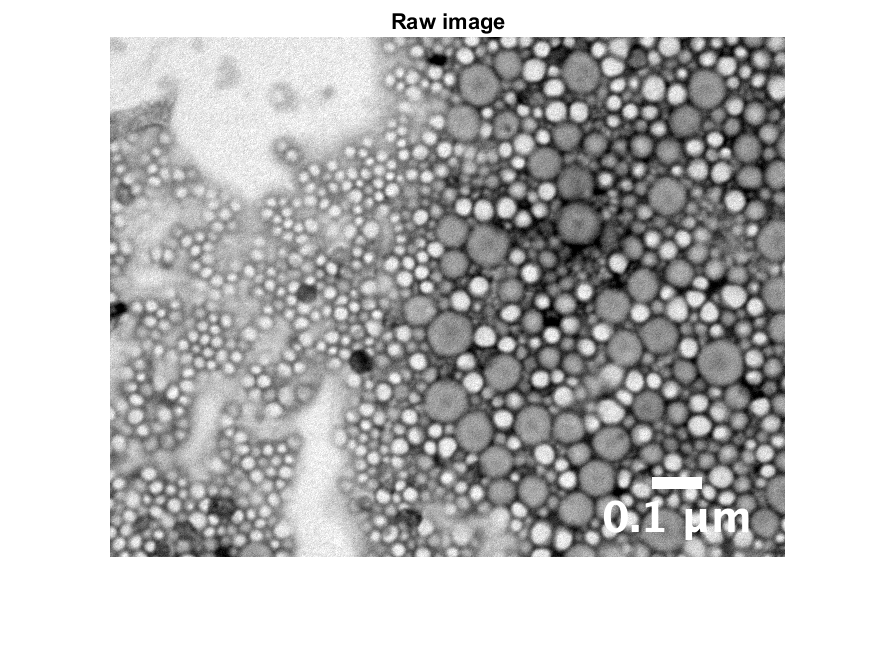

Supplement: Supplementary file 3 — Source Data [file 41467_2020_18603_MOESM3_ESM.zip › DLS_Plots_3_samples/Raw1.png]

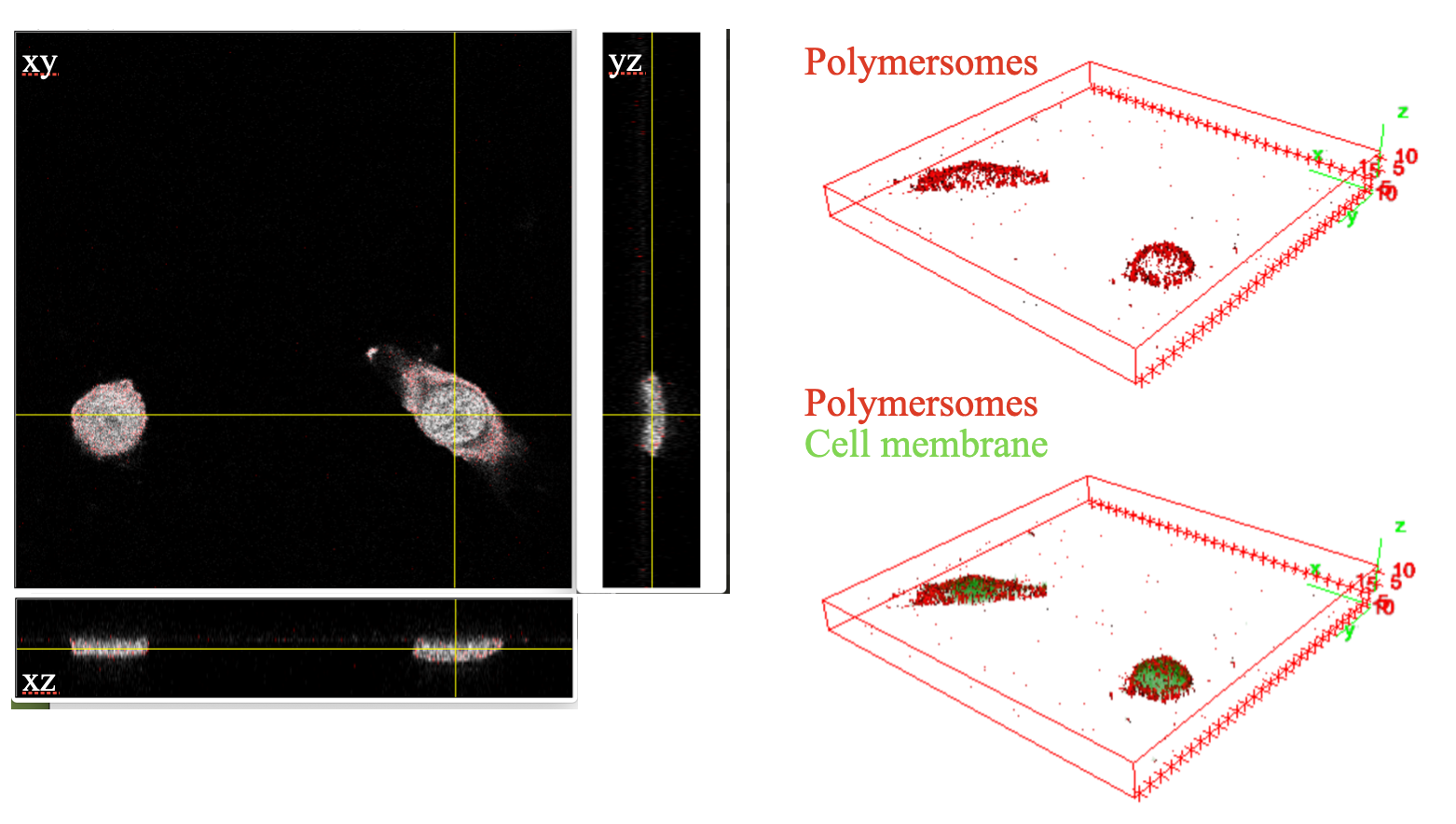

Supplement: Supplementary file 3 — Source Data [file 41467_2020_18603_MOESM3_ESM.zip › Fluorescence_microscopy/3D_viewer.png]

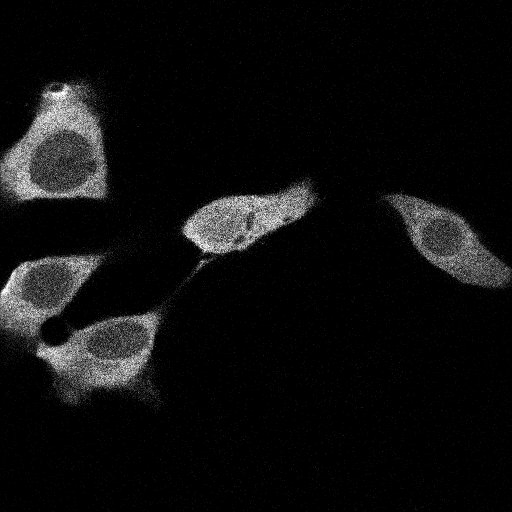

Supplement: Supplementary file 3 — Source Data [file 41467_2020_18603_MOESM3_ESM.zip › Fluorescence_microscopy/Cells 0%AP 60min -1.jpg]

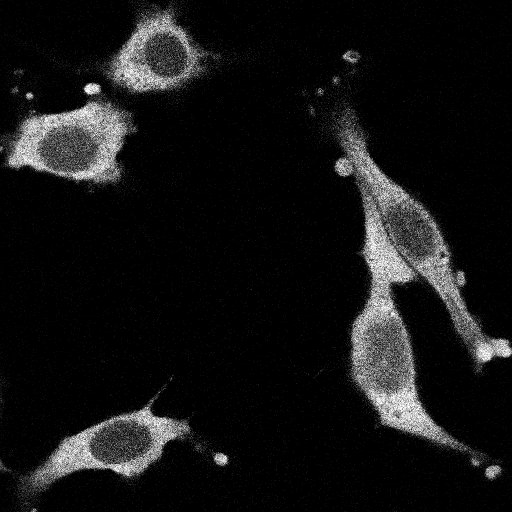

Supplement: Supplementary file 3 — Source Data [file 41467_2020_18603_MOESM3_ESM.zip › Fluorescence_microscopy/Cells 0.5%AP 60min .jpg]

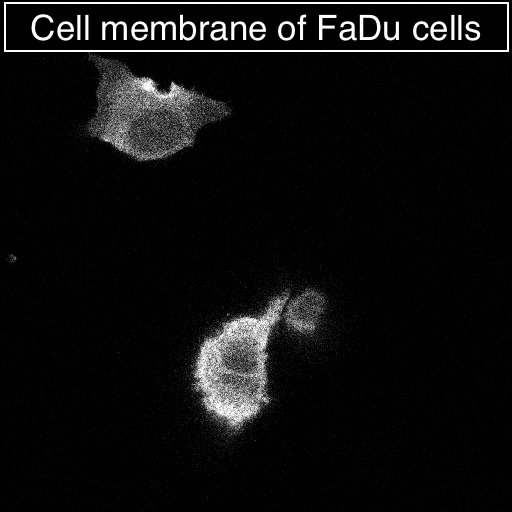

Supplement: Supplementary file 3 — Source Data [file 41467_2020_18603_MOESM3_ESM.zip › Fluorescence_microscopy/Cells 1%AP 60min .jpg]

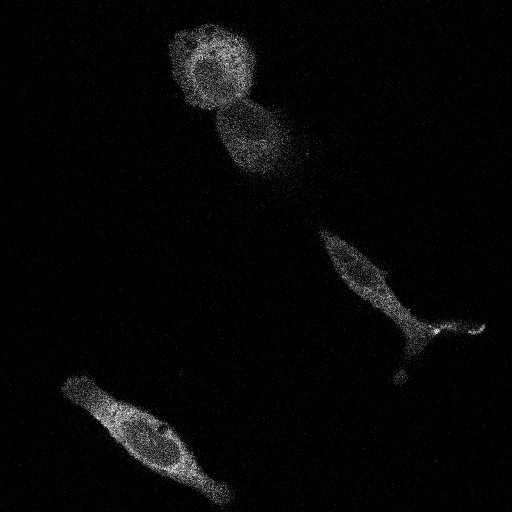

Supplement: Supplementary file 3 — Source Data [file 41467_2020_18603_MOESM3_ESM.zip › Fluorescence_microscopy/Cells 10%AP 60min .jpg]

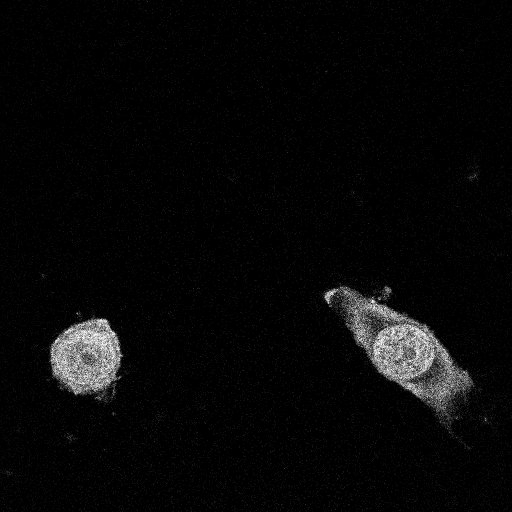

Supplement: Supplementary file 3 — Source Data [file 41467_2020_18603_MOESM3_ESM.zip › Fluorescence_microscopy/Cells2%AP 60min.jpg]

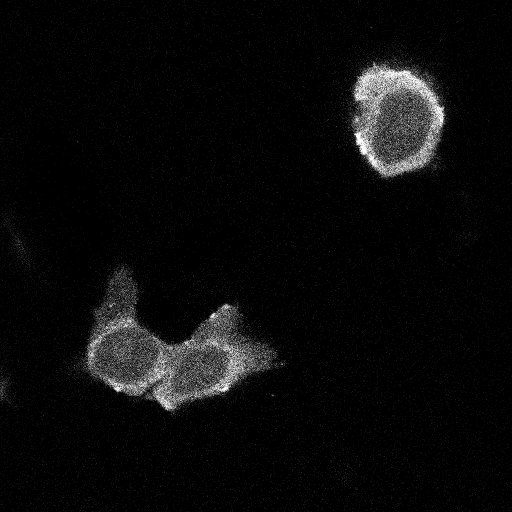

Supplement: Supplementary file 3 — Source Data [file 41467_2020_18603_MOESM3_ESM.zip › Fluorescence_microscopy/Cells5%AP 60min.jpg]

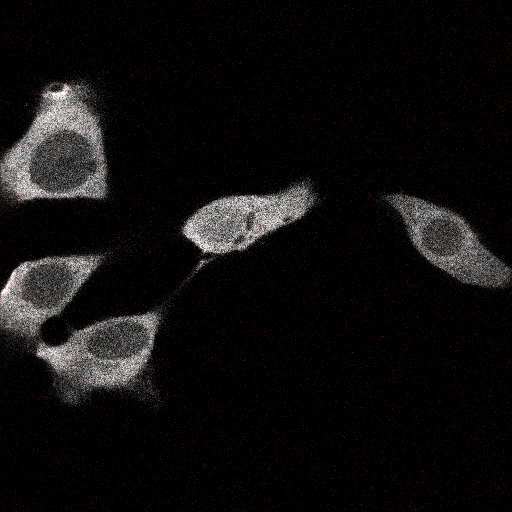

Supplement: Supplementary file 3 — Source Data [file 41467_2020_18603_MOESM3_ESM.zip › Fluorescence_microscopy/Composite0%AP 60min-1.jpg]

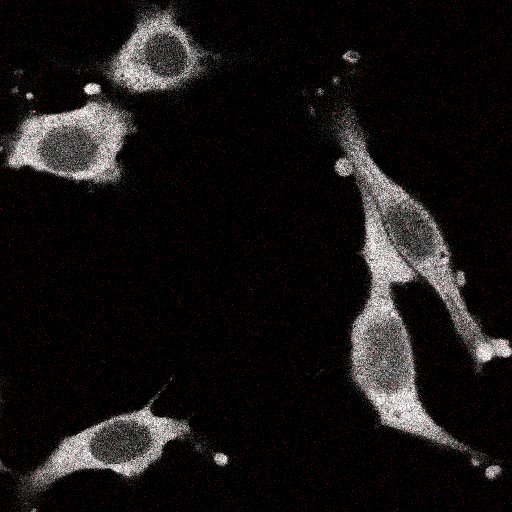

Supplement: Supplementary file 3 — Source Data [file 41467_2020_18603_MOESM3_ESM.zip › Fluorescence_microscopy/Composite0.5%AP 60min.jpg]

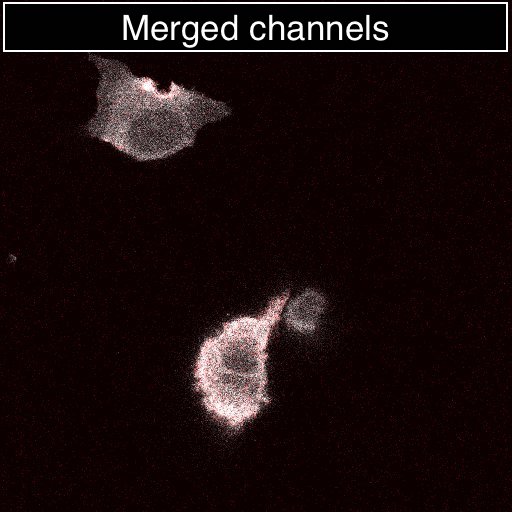

Supplement: Supplementary file 3 — Source Data [file 41467_2020_18603_MOESM3_ESM.zip › Fluorescence_microscopy/Composite1%AP 60min.jpg]

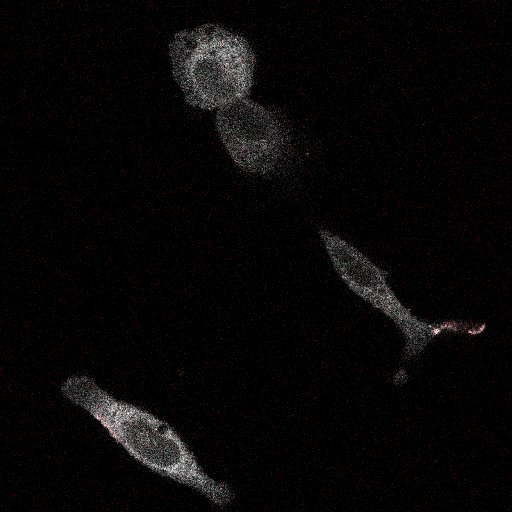

Supplement: Supplementary file 3 — Source Data [file 41467_2020_18603_MOESM3_ESM.zip › Fluorescence_microscopy/Composite10%AP 60min.jpg]

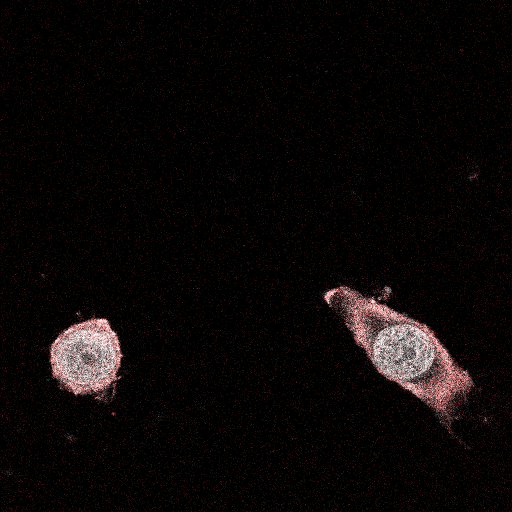

Supplement: Supplementary file 3 — Source Data [file 41467_2020_18603_MOESM3_ESM.zip › Fluorescence_microscopy/Composite2%AP 60min.jpg]

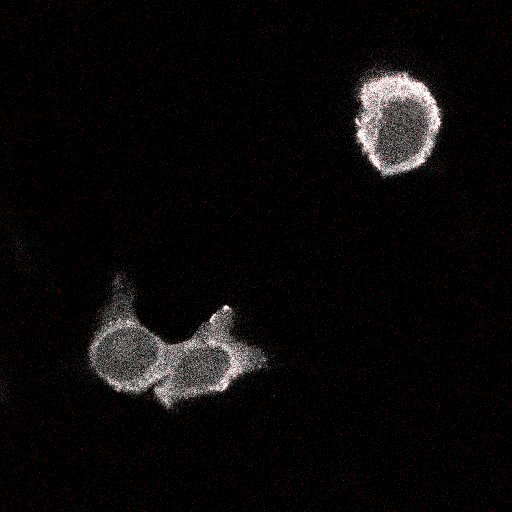

Supplement: Supplementary file 3 — Source Data [file 41467_2020_18603_MOESM3_ESM.zip › Fluorescence_microscopy/Composite5%AP 60min.jpg]

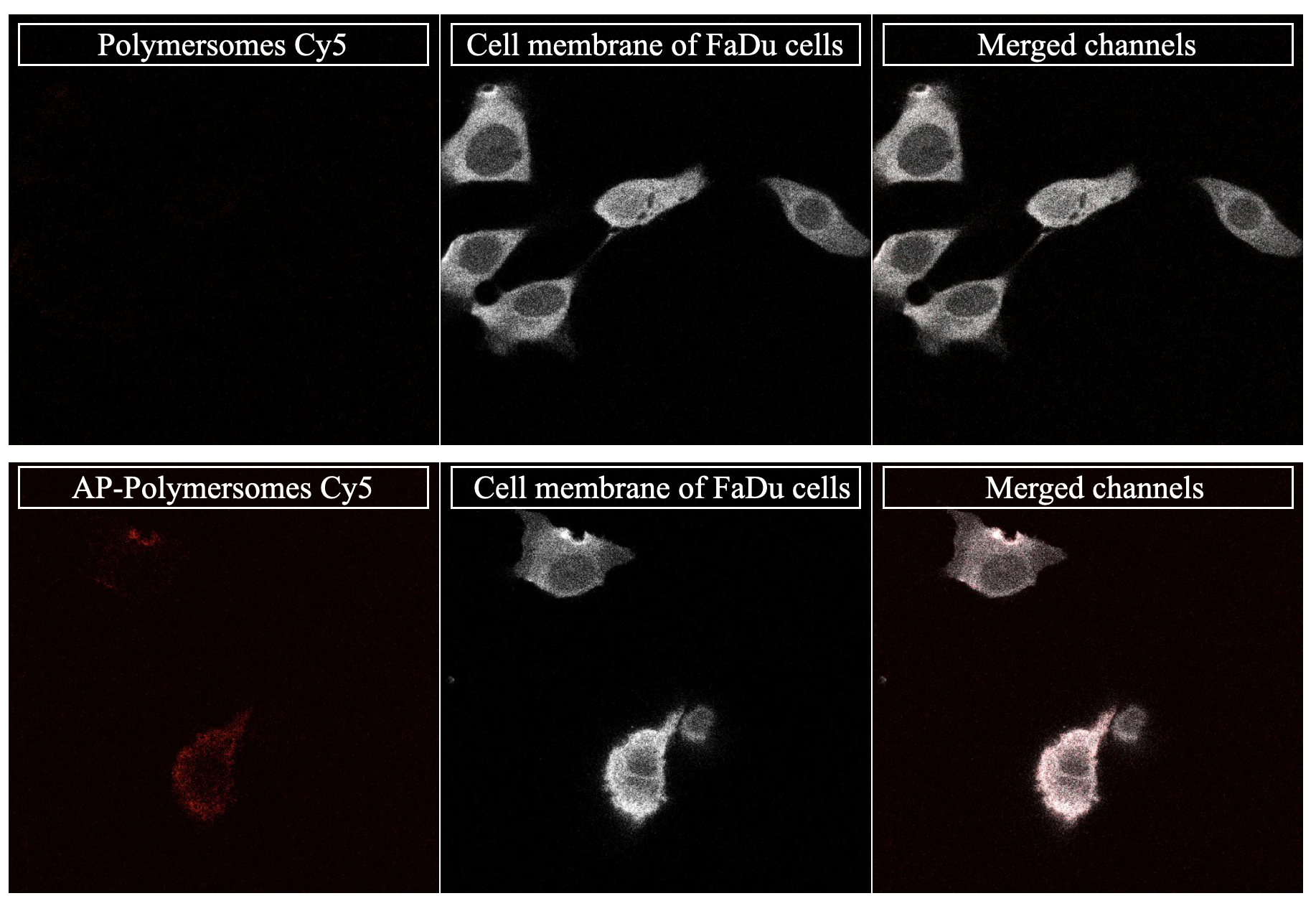

Supplement: Supplementary file 3 — Source Data [file 41467_2020_18603_MOESM3_ESM.zip › Fluorescence_microscopy/Fig 4b-1.png]

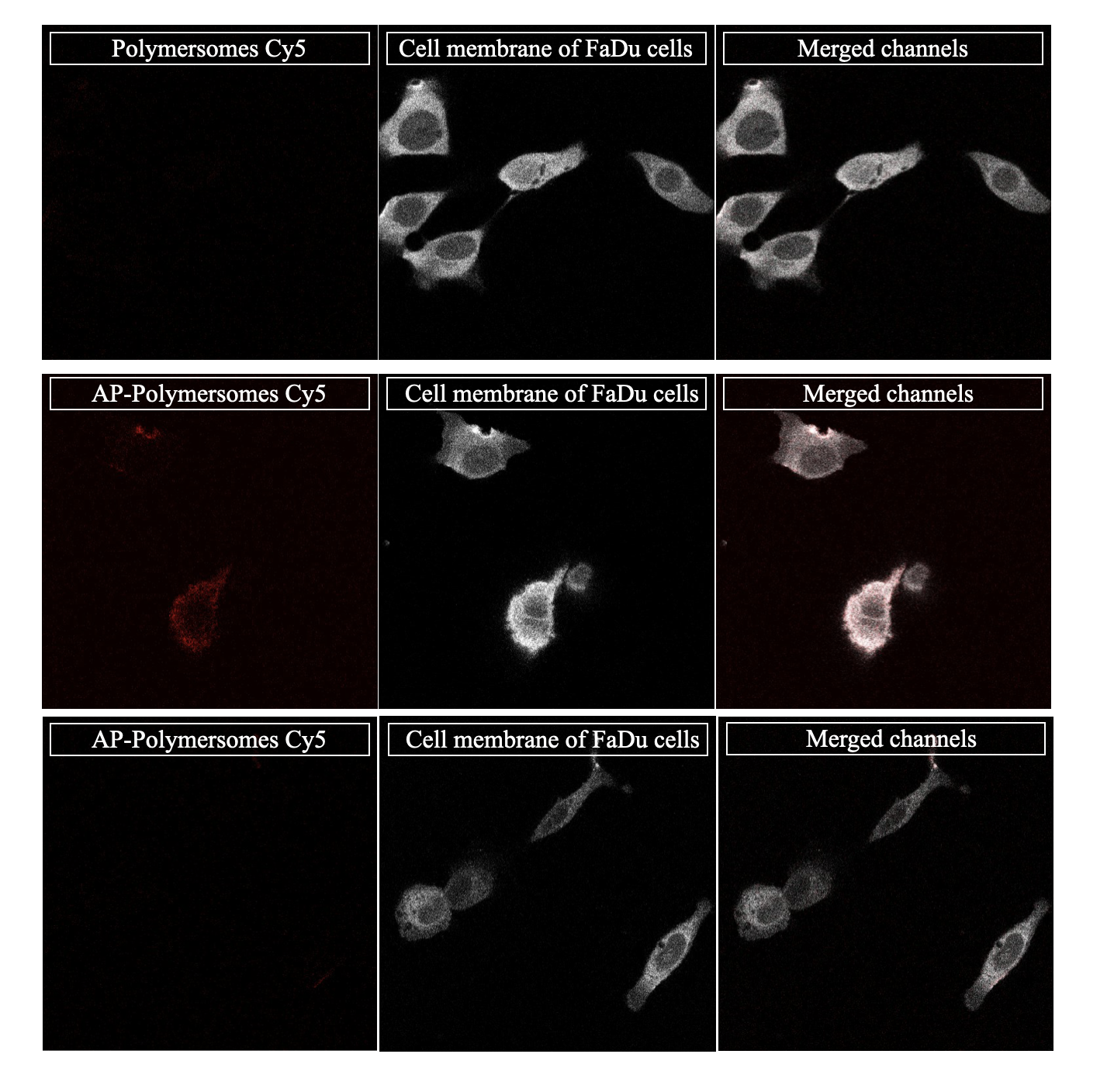

Supplement: Supplementary file 3 — Source Data [file 41467_2020_18603_MOESM3_ESM.zip › Fluorescence_microscopy/New Fig 4b.png]

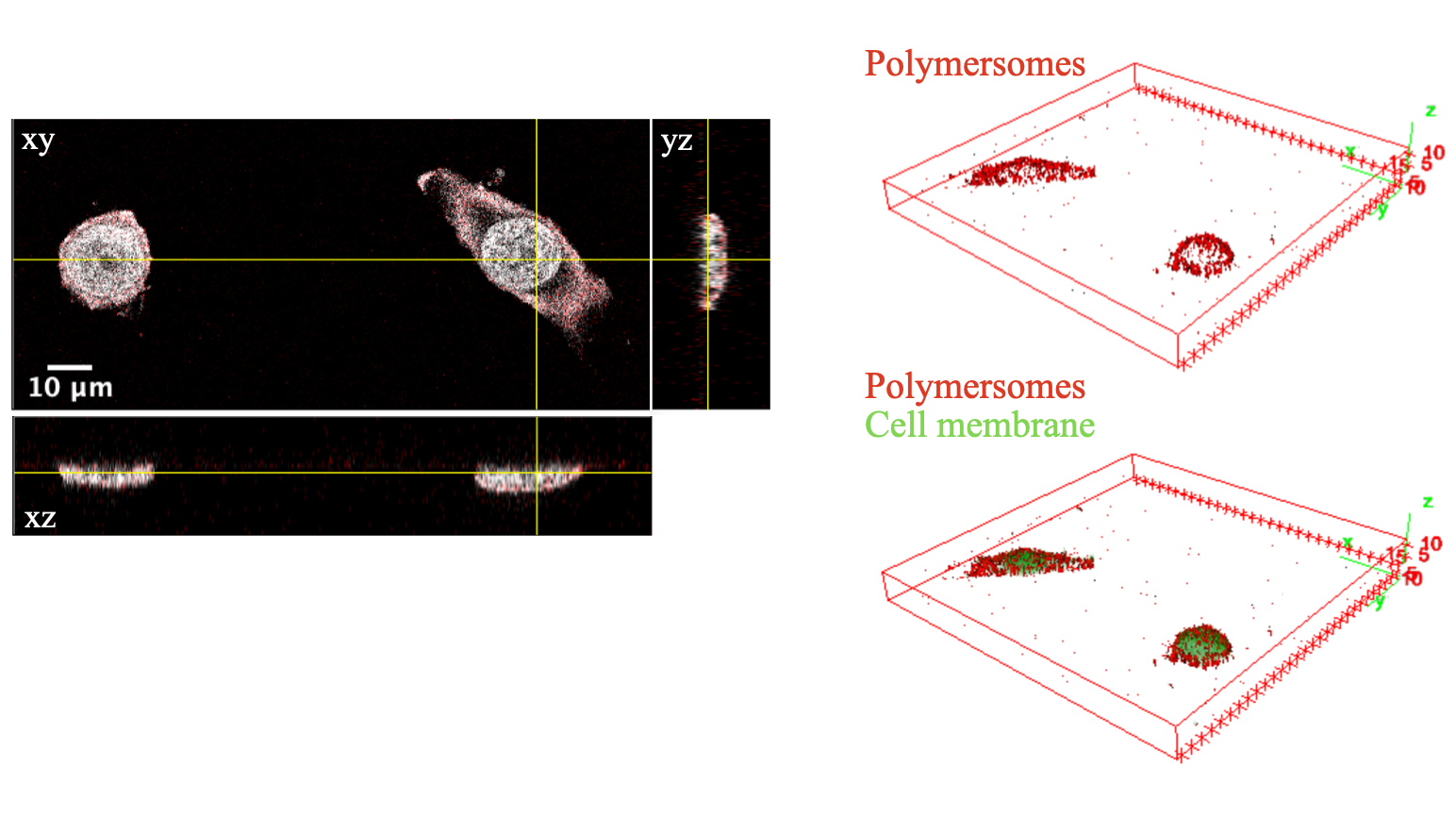

Supplement: Supplementary file 3 — Source Data [file 41467_2020_18603_MOESM3_ESM.zip › Fluorescence_microscopy/New_3D_image.png]

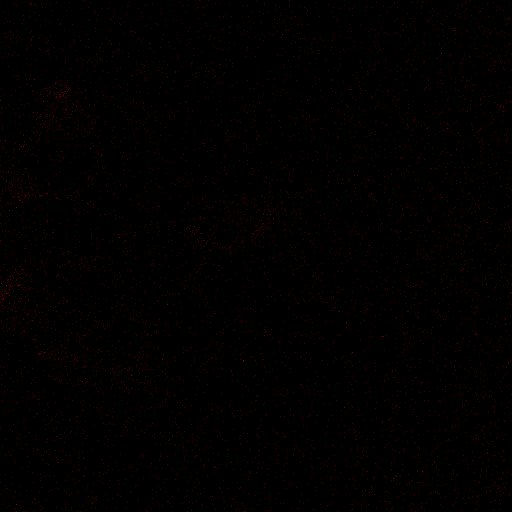

Supplement: Supplementary file 3 — Source Data [file 41467_2020_18603_MOESM3_ESM.zip › Fluorescence_microscopy/Psomes 0%AP 60min-1.jpg]

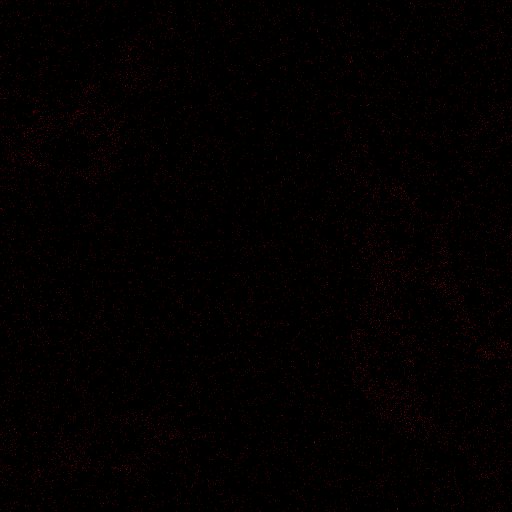

Supplement: Supplementary file 3 — Source Data [file 41467_2020_18603_MOESM3_ESM.zip › Fluorescence_microscopy/Psomes 0.5%AP 60min.jpg]

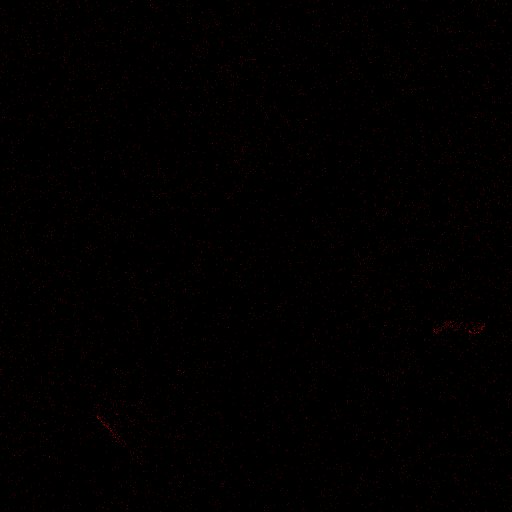

Supplement: Supplementary file 3 — Source Data [file 41467_2020_18603_MOESM3_ESM.zip › Fluorescence_microscopy/Psomes 10%AP 60min.jpg]

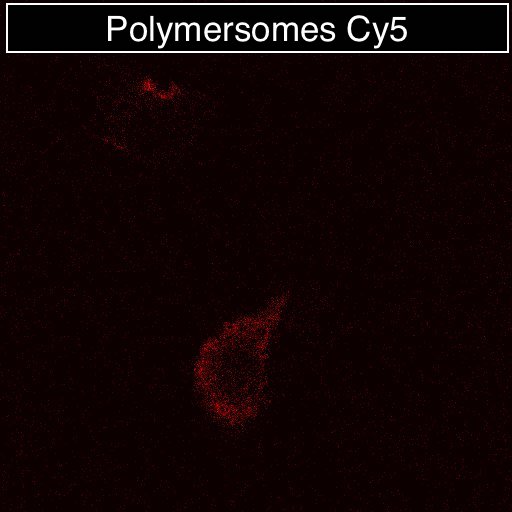

Supplement: Supplementary file 3 — Source Data [file 41467_2020_18603_MOESM3_ESM.zip › Fluorescence_microscopy/Psomes1%AP 60min.jpg]

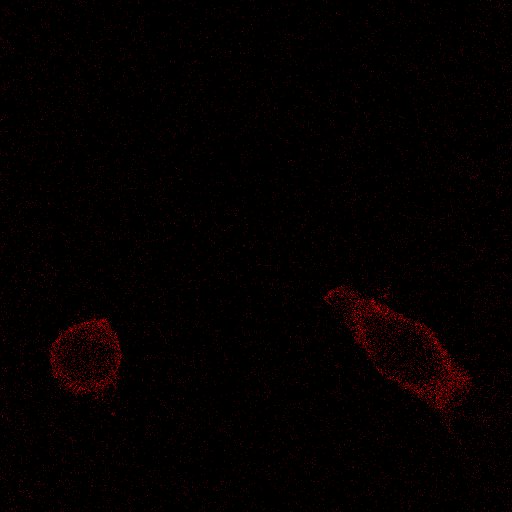

Supplement: Supplementary file 3 — Source Data [file 41467_2020_18603_MOESM3_ESM.zip › Fluorescence_microscopy/Psomes2%AP 60min.jpg]

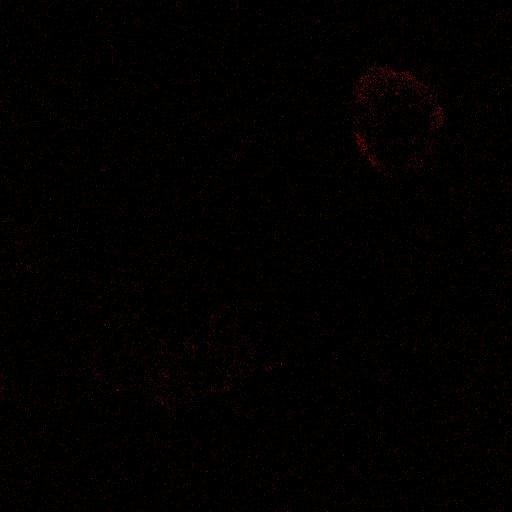

Supplement: Supplementary file 3 — Source Data [file 41467_2020_18603_MOESM3_ESM.zip › Fluorescence_microscopy/Psomes5%AP 60min.jpg]

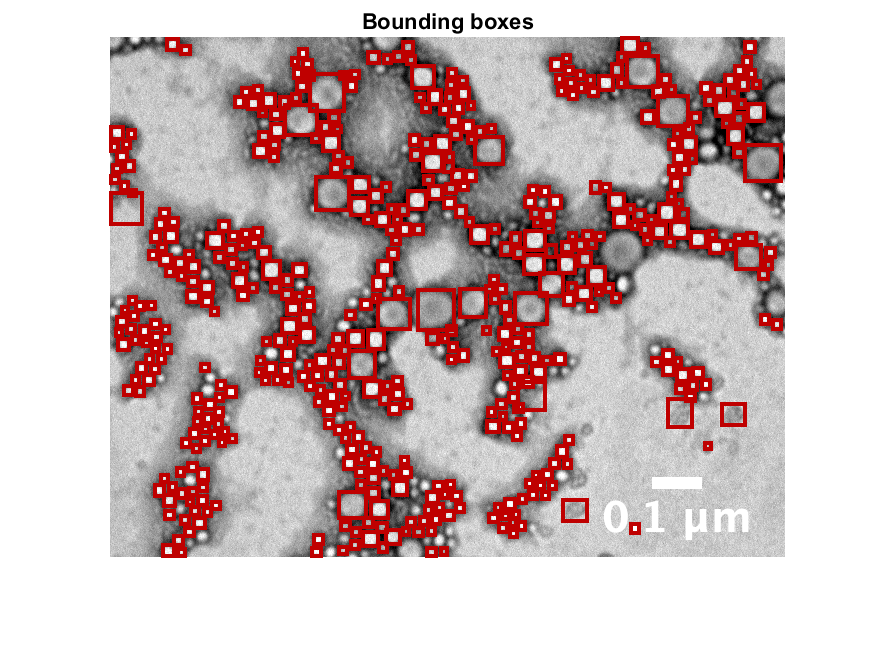

Supplement: Supplementary file 3 — Source Data [file 41467_2020_18603_MOESM3_ESM.zip › TEM_analyzed/BB0.5.png]

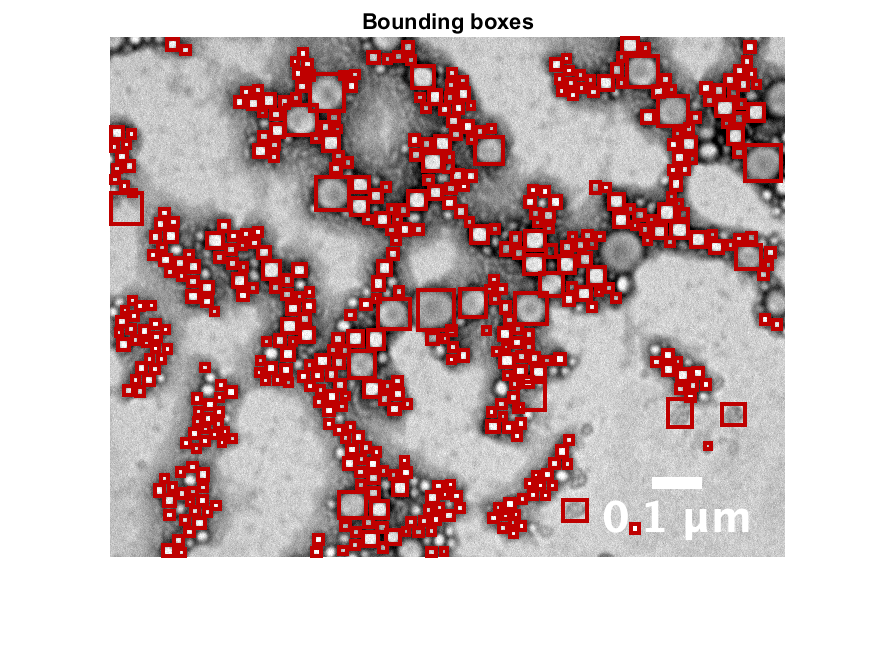

Supplement: Supplementary file 3 — Source Data [file 41467_2020_18603_MOESM3_ESM.zip › TEM_analyzed/BB0.5.tif]

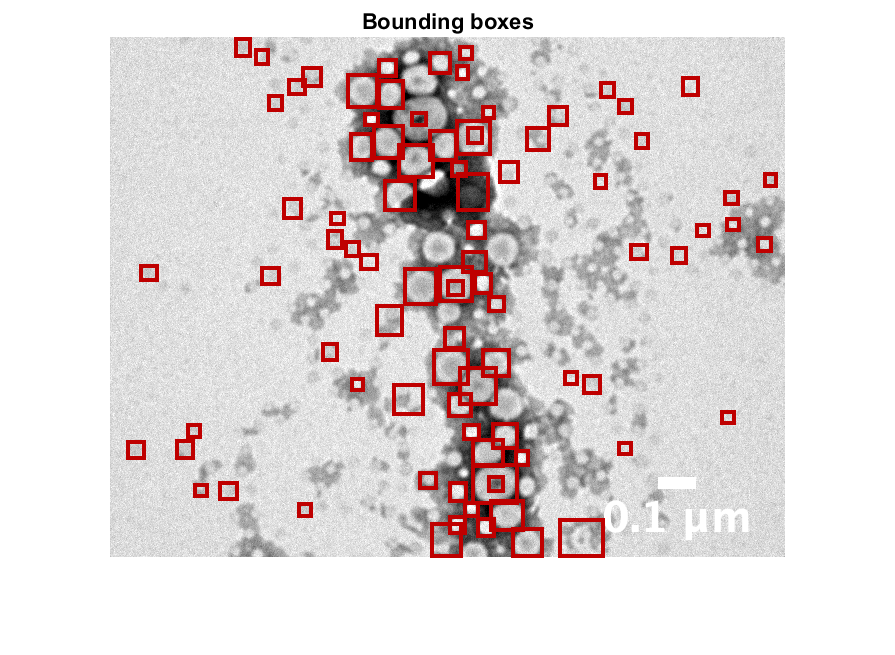

Supplement: Supplementary file 3 — Source Data [file 41467_2020_18603_MOESM3_ESM.zip › TEM_analyzed/BB0.tif]

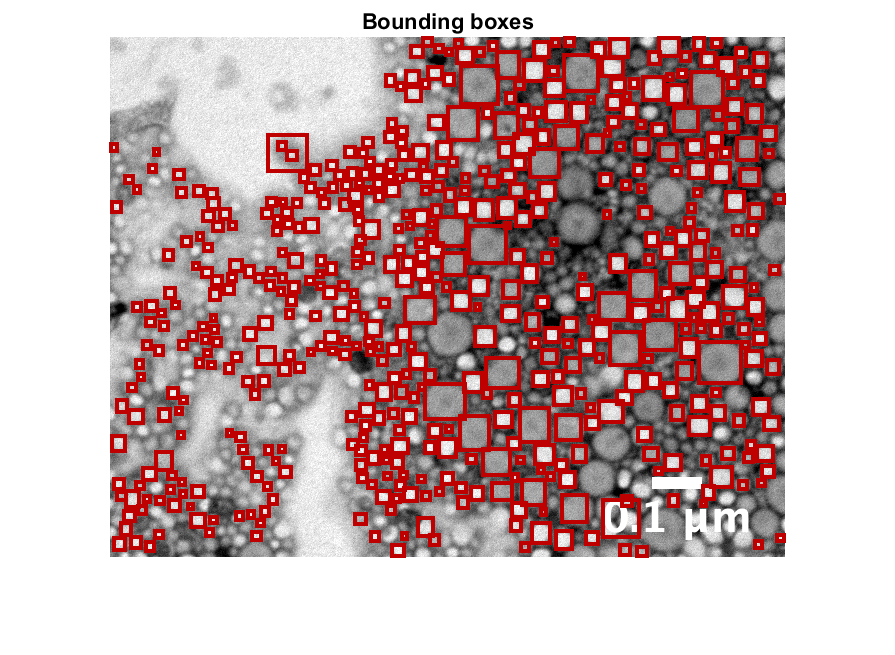

Supplement: Supplementary file 3 — Source Data [file 41467_2020_18603_MOESM3_ESM.zip › TEM_analyzed/BB1.tif]

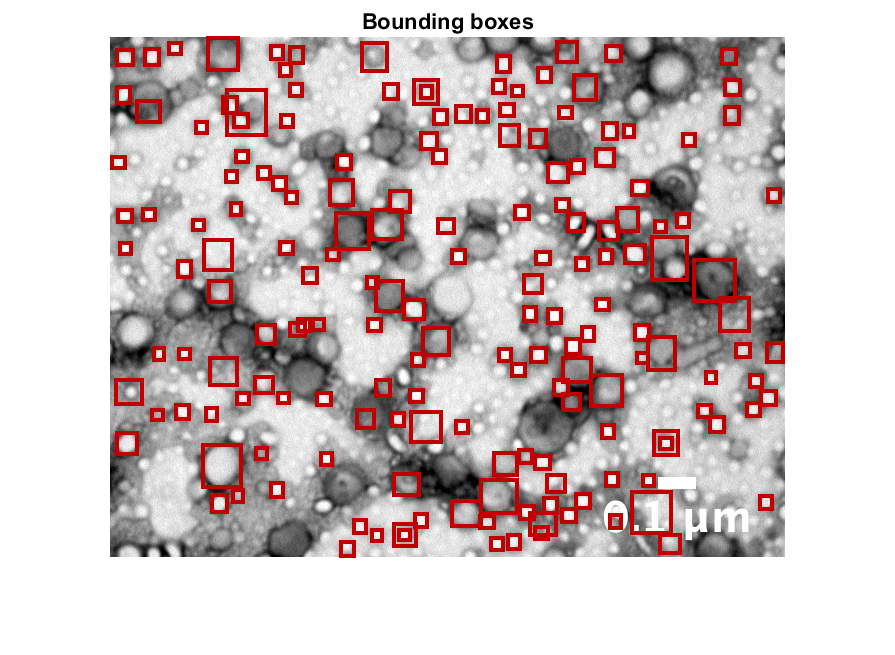

Supplement: Supplementary file 3 — Source Data [file 41467_2020_18603_MOESM3_ESM.zip › TEM_analyzed/BB10.tif]

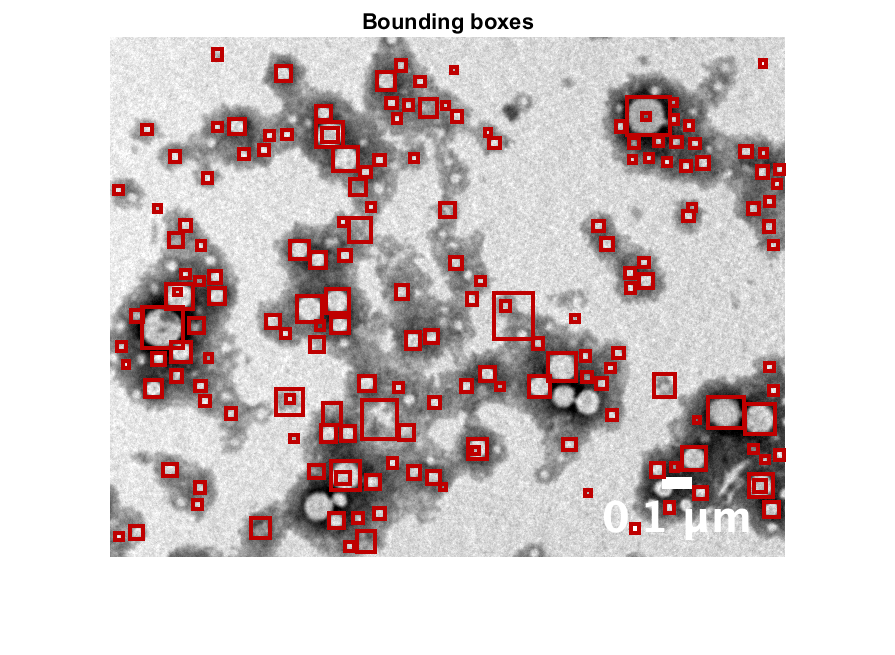

Supplement: Supplementary file 3 — Source Data [file 41467_2020_18603_MOESM3_ESM.zip › TEM_analyzed/BB5.tif]

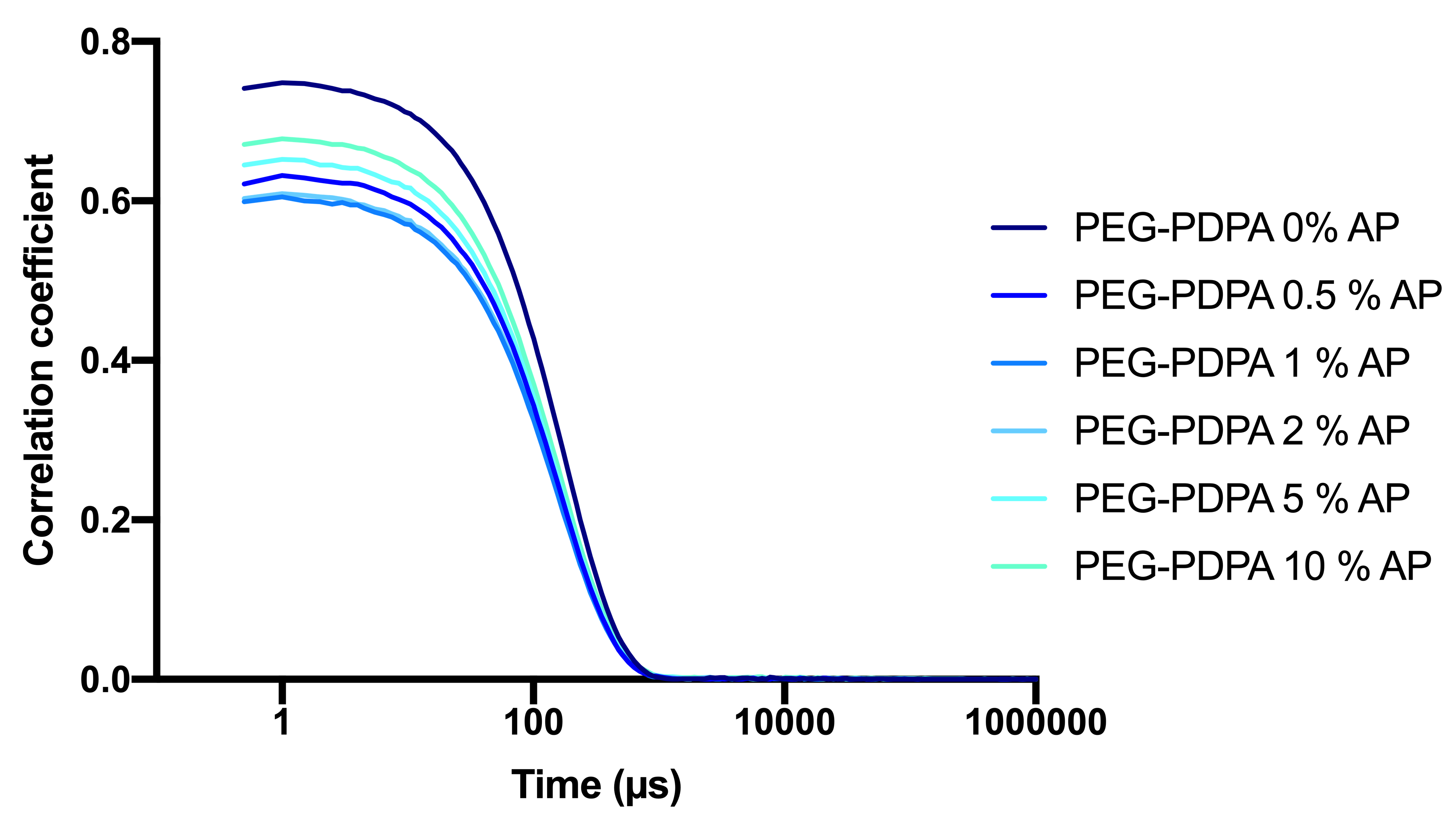

Supplement: Supplementary file 3 — Source Data [file 41467_2020_18603_MOESM3_ESM.zip › TEM_analyzed/Correlograms.png]

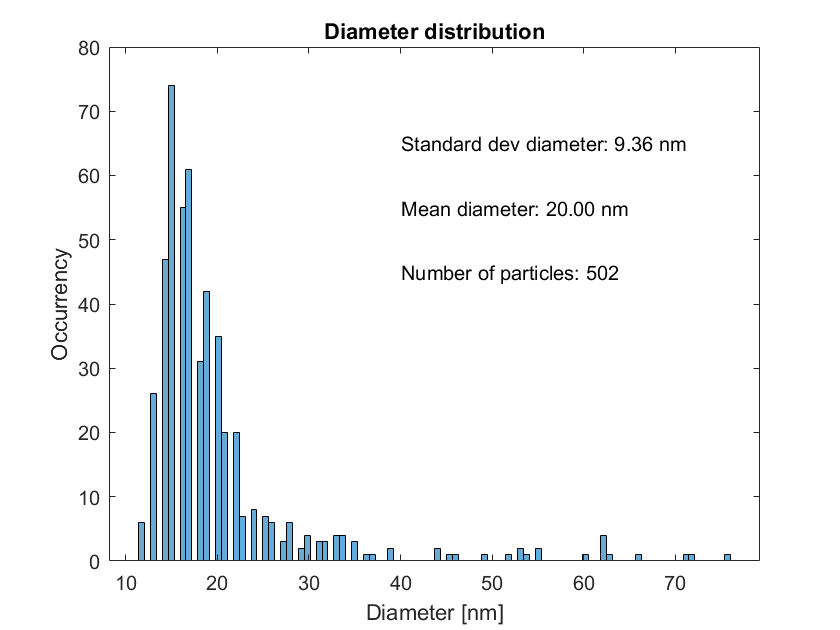

Supplement: Supplementary file 3 — Source Data [file 41467_2020_18603_MOESM3_ESM.zip › TEM_analyzed/Hist0.5.png]

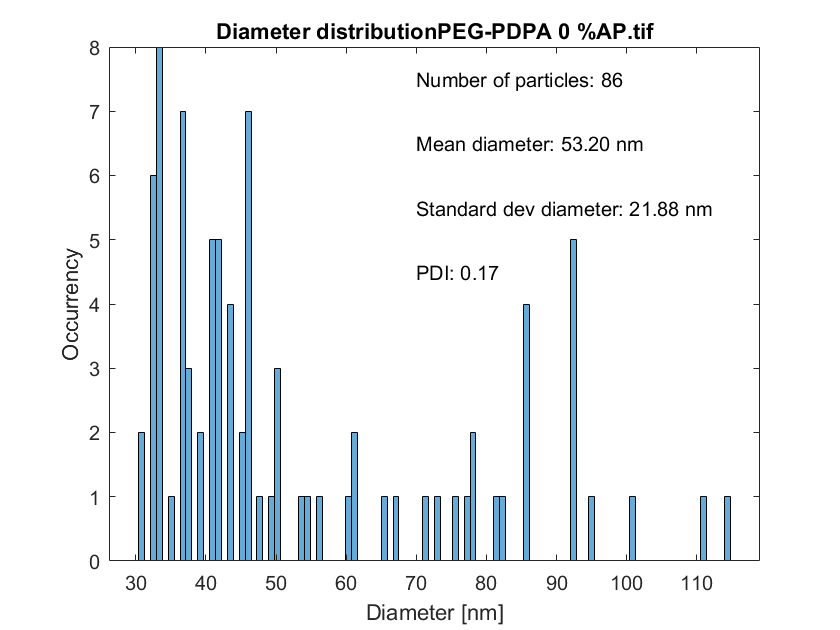

Supplement: Supplementary file 3 — Source Data [file 41467_2020_18603_MOESM3_ESM.zip › TEM_analyzed/Hist0.png]

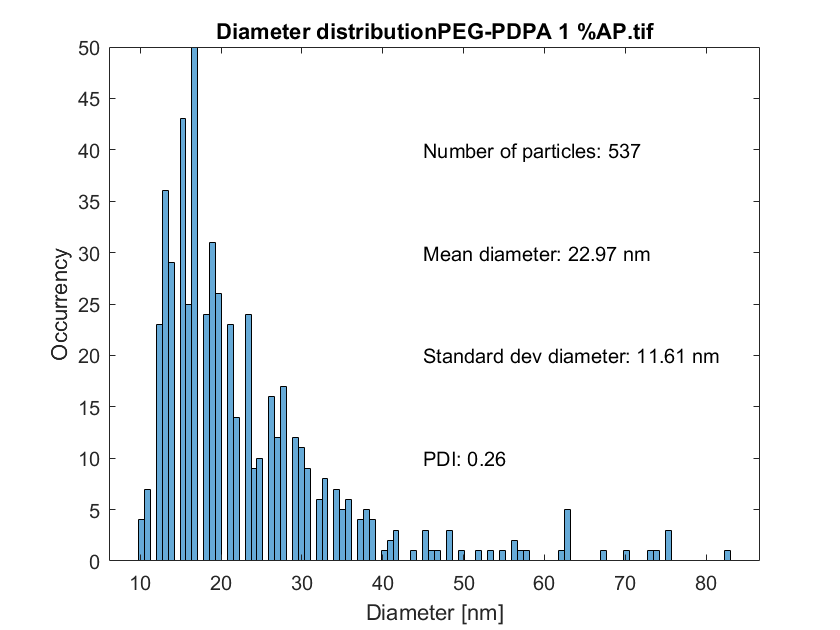

Supplement: Supplementary file 3 — Source Data [file 41467_2020_18603_MOESM3_ESM.zip › TEM_analyzed/Hist1.png]

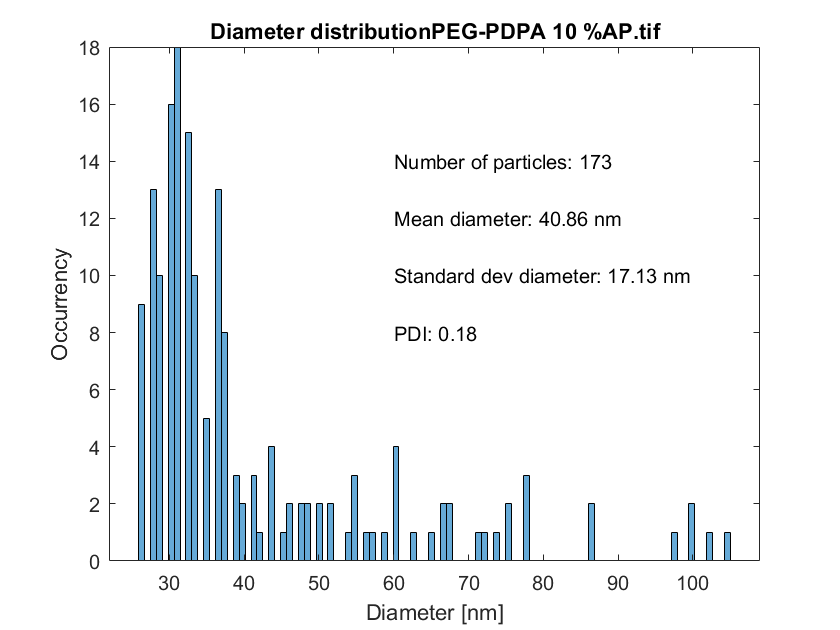

Supplement: Supplementary file 3 — Source Data [file 41467_2020_18603_MOESM3_ESM.zip › TEM_analyzed/Hist10.png]

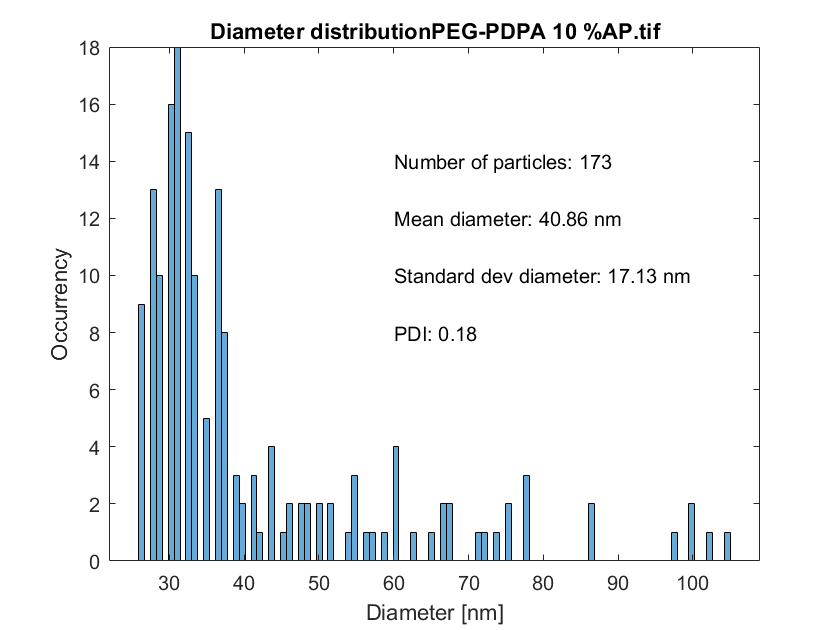

Supplement: Supplementary file 3 — Source Data [file 41467_2020_18603_MOESM3_ESM.zip › TEM_analyzed/Hist10.tif]

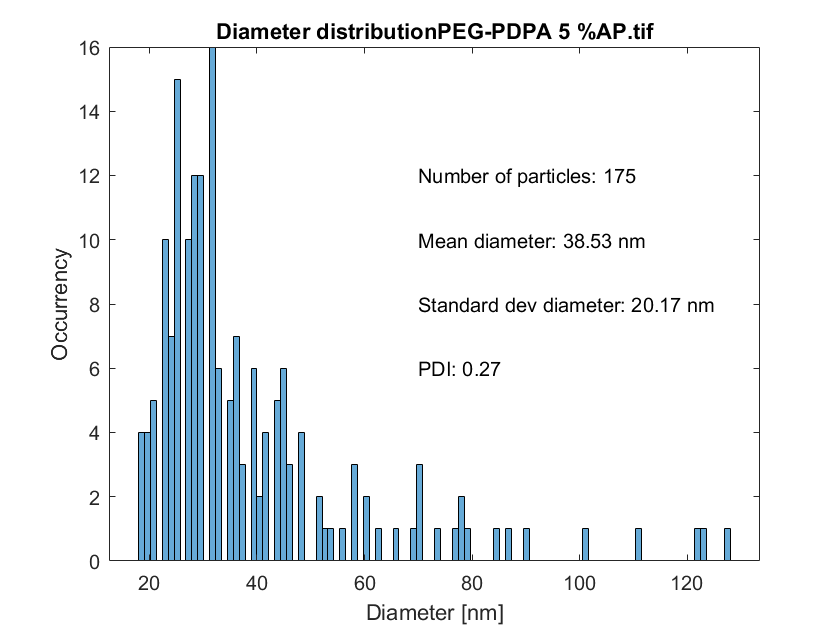

Supplement: Supplementary file 3 — Source Data [file 41467_2020_18603_MOESM3_ESM.zip › TEM_analyzed/Hist5.png]

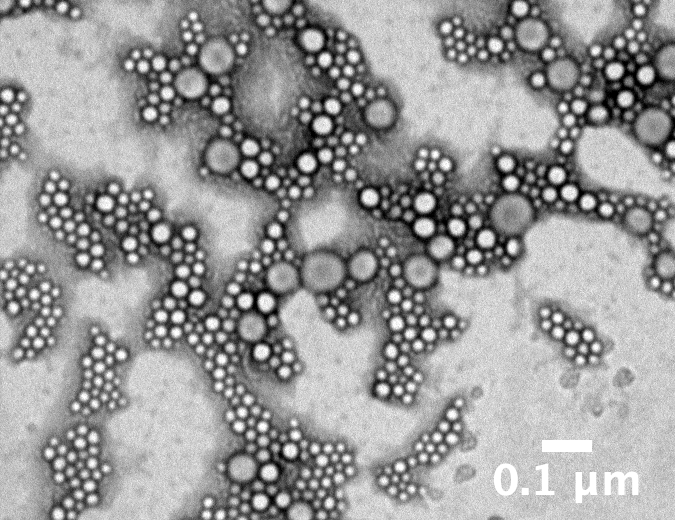

Supplement: Supplementary file 3 — Source Data [file 41467_2020_18603_MOESM3_ESM.zip › TEM_analyzed/Raw0.5.png]

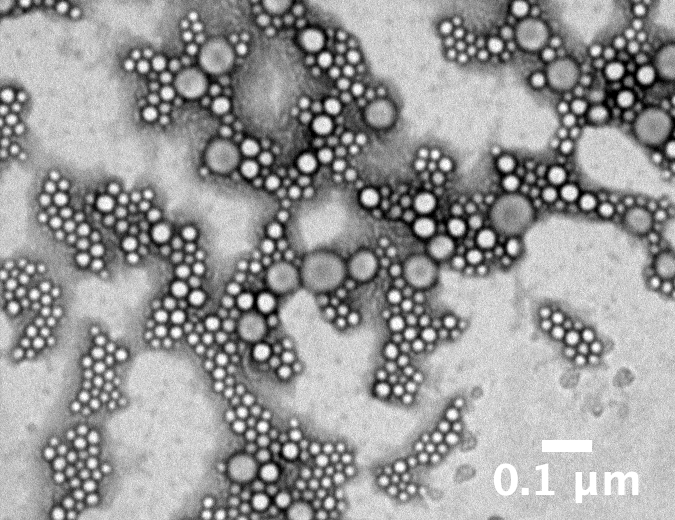

Supplement: Supplementary file 3 — Source Data [file 41467_2020_18603_MOESM3_ESM.zip › TEM_analyzed/Raw0.5.tiff]

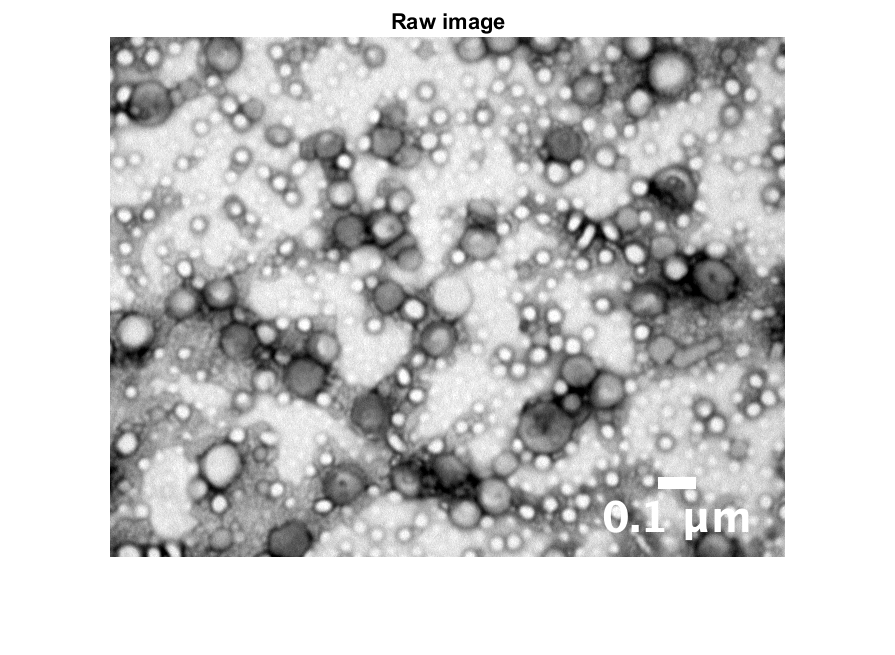

Supplement: Supplementary file 3 — Source Data [file 41467_2020_18603_MOESM3_ESM.zip › TEM_analyzed/Raw10.png]

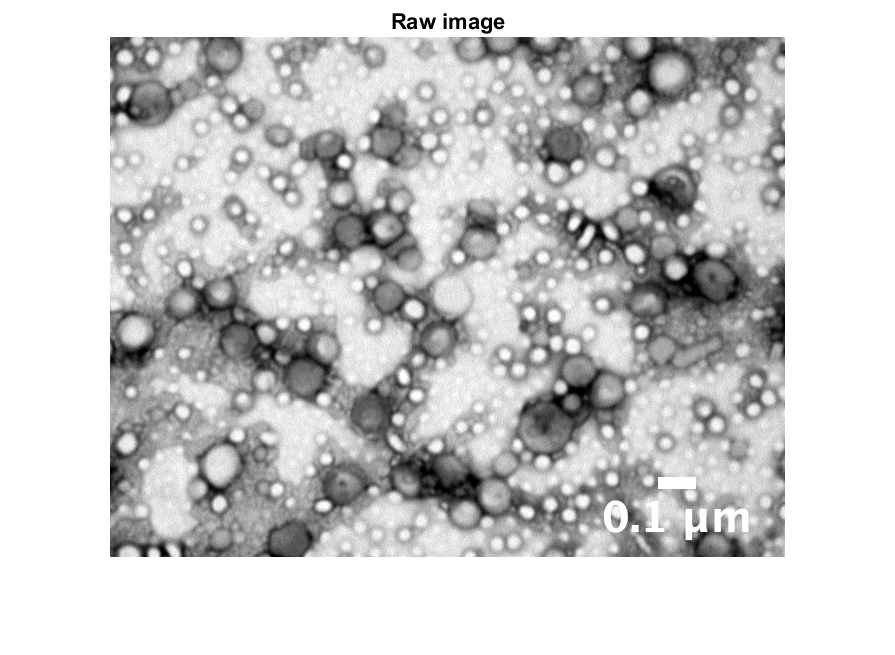

Supplement: Supplementary file 3 — Source Data [file 41467_2020_18603_MOESM3_ESM.zip › TEM_analyzed/Raw10.tif]

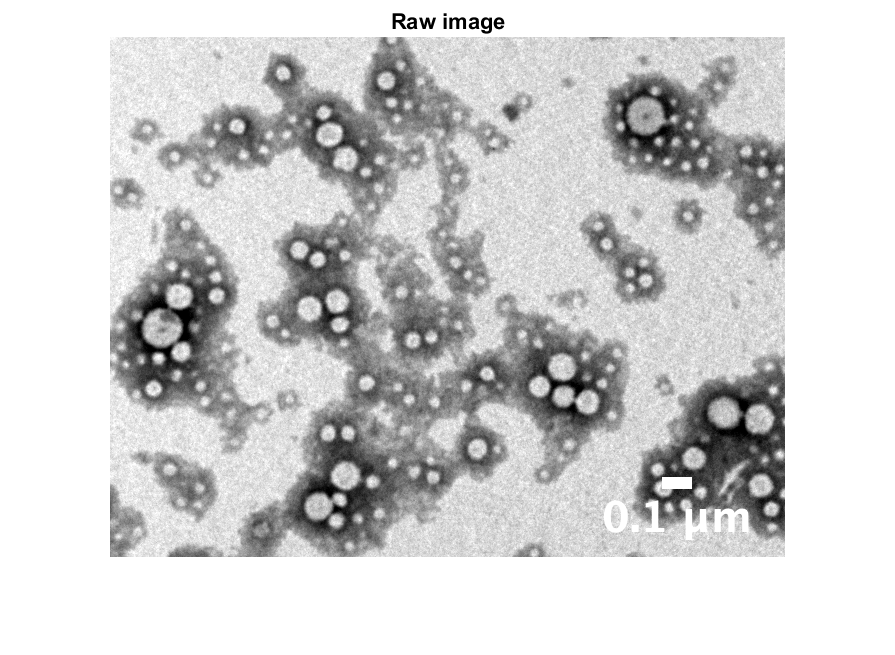

Supplement: Supplementary file 3 — Source Data [file 41467_2020_18603_MOESM3_ESM.zip › TEM_analyzed/Raw5.png]

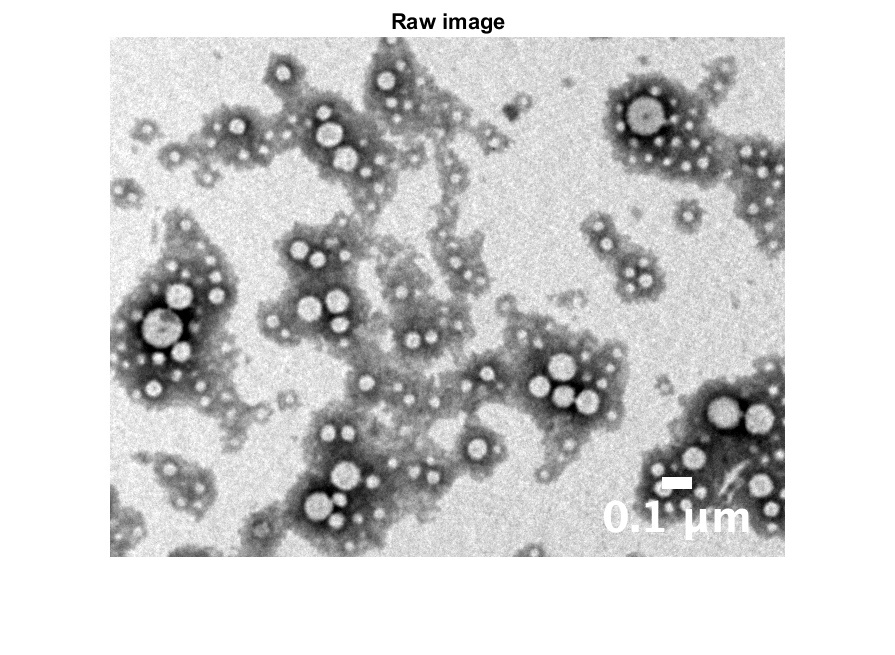

Supplement: Supplementary file 3 — Source Data [file 41467_2020_18603_MOESM3_ESM.zip › TEM_analyzed/Raw5.tif]

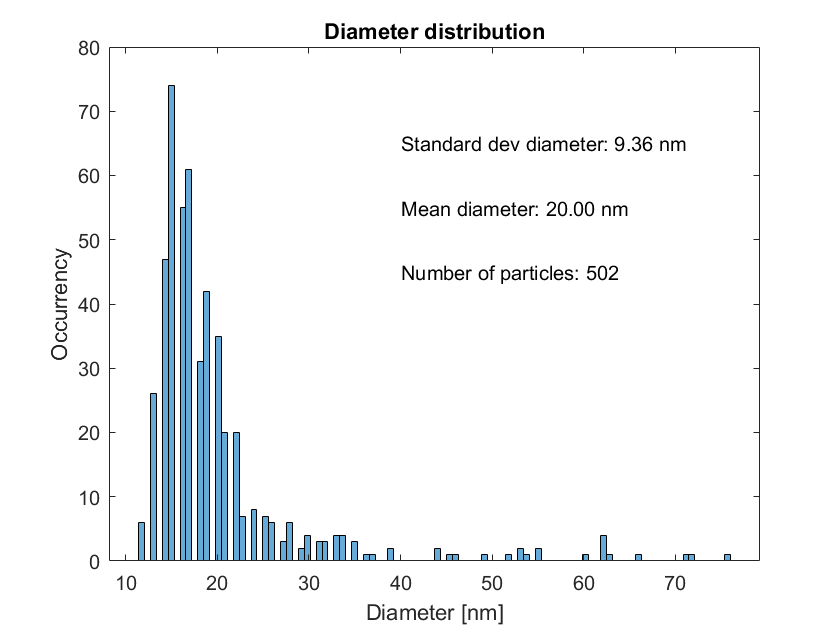

Supplement: Supplementary file 3 — Source Data [file 41467_2020_18603_MOESM3_ESM.zip › TEM_analyzed/tif/Hist0.5.tif]

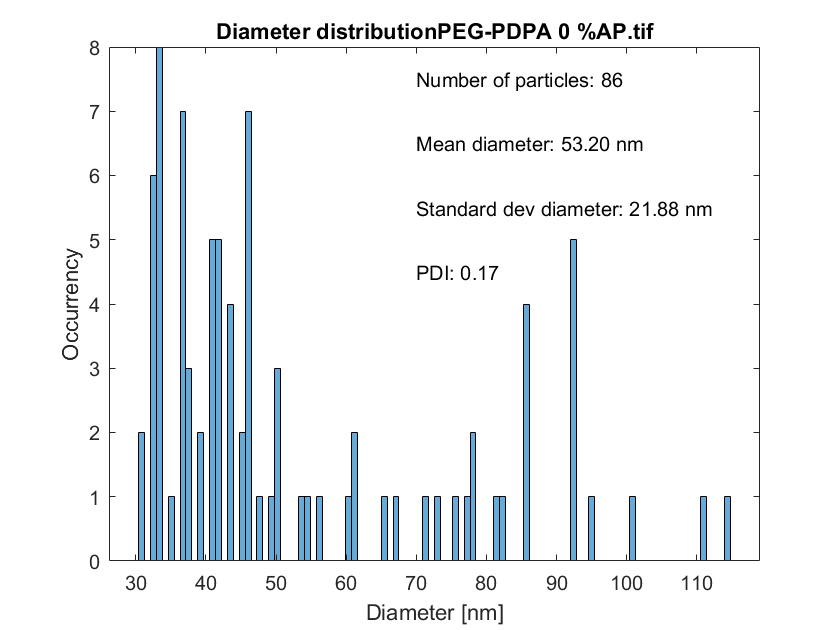

Supplement: Supplementary file 3 — Source Data [file 41467_2020_18603_MOESM3_ESM.zip › TEM_analyzed/tif/Hist0.tif]

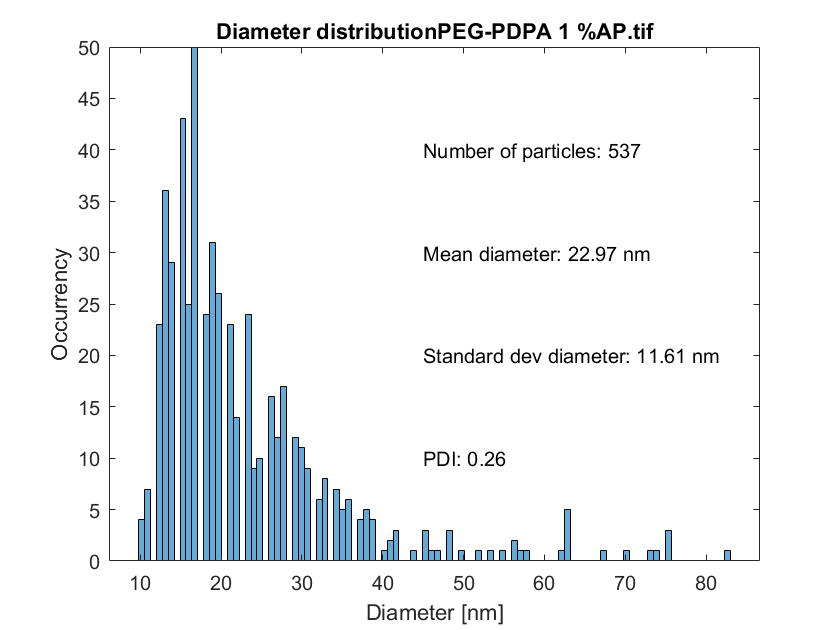

Supplement: Supplementary file 3 — Source Data [file 41467_2020_18603_MOESM3_ESM.zip › TEM_analyzed/tif/Hist1.tif]

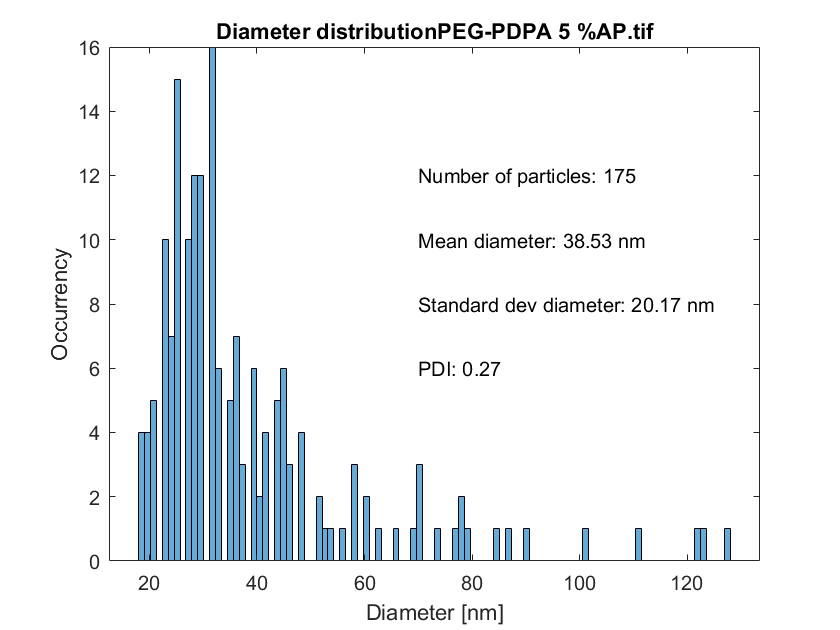

Supplement: Supplementary file 3 — Source Data [file 41467_2020_18603_MOESM3_ESM.zip › TEM_analyzed/tif/Hist5.tif]

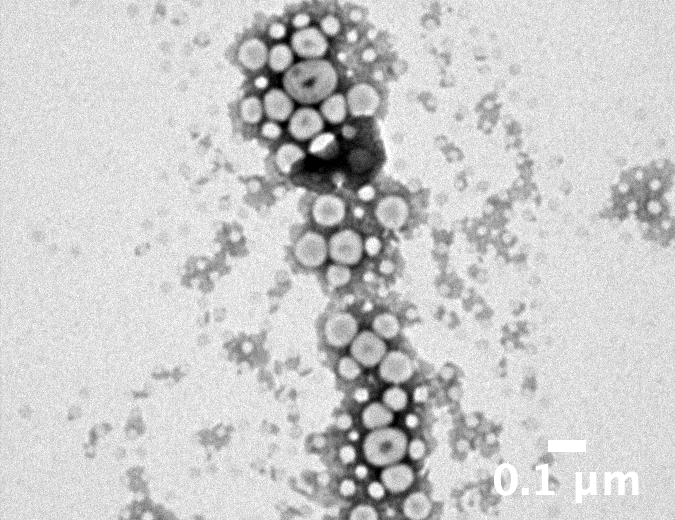

Supplement: Supplementary file 3 — Source Data [file 41467_2020_18603_MOESM3_ESM.zip › TEM_data_size_polymersomes_(not shown)/PEG-PDPA 0 %AP.tif]

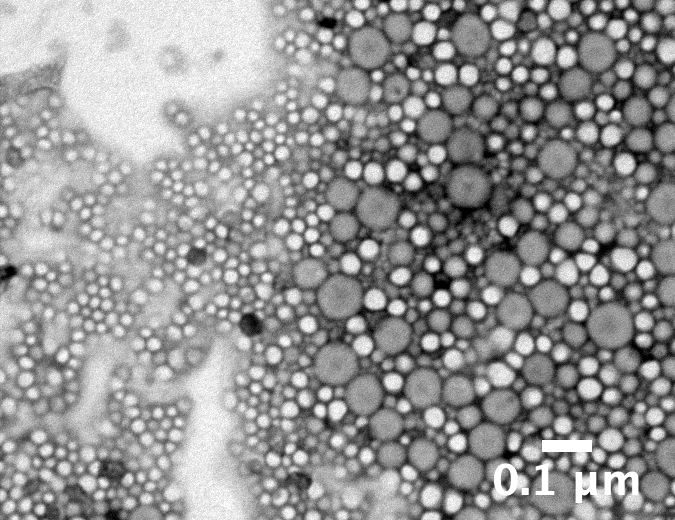

Supplement: Supplementary file 3 — Source Data [file 41467_2020_18603_MOESM3_ESM.zip › TEM_data_size_polymersomes_(not shown)/PEG-PDPA 1 %AP.tif]

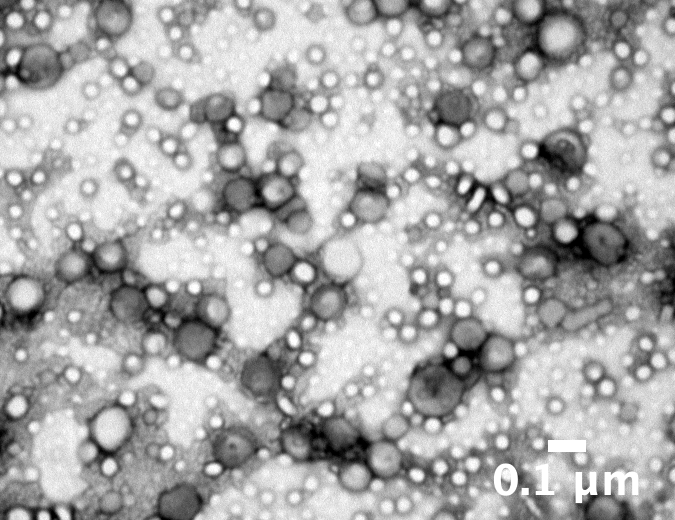

Supplement: Supplementary file 3 — Source Data [file 41467_2020_18603_MOESM3_ESM.zip › TEM_data_size_polymersomes_(not shown)/PEG-PDPA 10 %AP.tif]

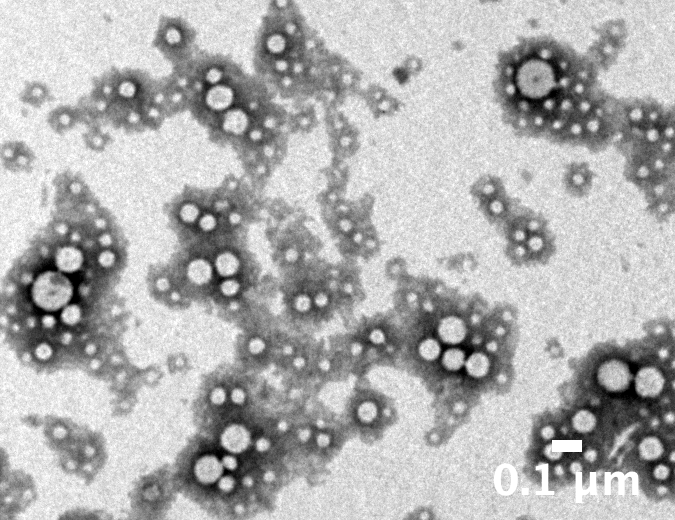

Supplement: Supplementary file 3 — Source Data [file 41467_2020_18603_MOESM3_ESM.zip › TEM_data_size_polymersomes_(not shown)/PEG-PDPA 5 %AP.tif]
